# Comparative metabolomics study on the secondary metabolites of the red alga, *Corallina officinalis* and its associated endosymbiotic fungi†

**DOI:** 10.1039/d4ra01055h

**Published:** 2024-06-20

**Authors:** Sherif M. Shama, Ahmed M. Elissawy, Mohamed A. Salem, Fadia S. Youssef, Mohamed S. Elnaggar, Hesham R. El-Seedi, Shaden A. M. Khalifa, Khaled Briki, Dalia Ibrahim Hamdan, Abdel Nasser B. Singab

**Affiliations:** a Department of Pharmacognosy and Natural Products, Faculty of Pharmacy, Menoufia University Shibin Elkom 32511 Egypt dean@pharma.asu.edu.eg; b Department of Pharmacognosy, Faculty of Pharmacy, Ain-Shams University Cairo 11566 Egypt; c Center of Drug Discovery Research and Development, Ain-Shams University Cairo 11566 Egypt; d Chemistry Department, Faculty of Science, Islamic University of Madinah P. O. Box: 170 Madinah 42351 Saudi Arabia; e International Research Center for Food Nutrition and Safety, Jiangsu University Zhenjiang 212013 China; f Psychiatry and Neurology Department, Capio Saint Göran's Hospital Sankt Göransplan 1 112 19 Stockholm Sweden; g Laboratory of Organic Chemistry and Natural Substance, University Ziane Achour Djelfa Algeria

## Abstract

Marine endosymbionts have gained remarkable interest in the last three decades in terms of natural products (NPs) isolated thereof, emphasizing the chemical correlations with those isolated from the host marine organism. The current study aimed to conduct comparative metabolic profiling of the marine red algae *Corallina officinalis*, and three fungal endosymbionts isolated from its inner tissues namely, *Aspergillus nidulans*, *A. flavipes* and *A. flavus*. The ethyl acetate (EtOAc) extracts of the host organism as well as the isolated endosymbionts were analyzed using ultra-high performance liquid chromatography coupled to high resolution tandem mass spectrometry (UHPLC-MS/MS)in both positive and negative ion modes, applying both full scan (FS) and all ion fragmentation (AIF) modes. Extensive interpretation of the LC-MS/MS spectra had led to the identification of 76 metabolites belonging to different phytochemical classes including alkaloids, polyketides, sesquiterpenes, butyrolactones, peptides, fatty acids, isocoumarins, quinones, among others. Metabolites were tentatively identified by comparing the accurate mass and fragmentation pattern with metabolites previously reported in the literature, as well as bioinformatics analysis using GNPS. A relationship between the host *C. officinalis* and its endophytes (*A. flavus*, *A. nidulans*, and *A. flavipes*) was discovered. *C. officinalis* shares common metabolites with at least one of the three endosymbiotic fungi. Some metabolites have been identified in endophytes and do not exist in their host. Multivariate analysis (MVA) revealed discrimination of *A. flavipes* from *Corallina officinalis* and other associated endophytic *Aspergillus* fungi (*A. flavus* and *A. nidulans*).

## Abstract 2

HR-UPLC-MS-MS aided metabolomic analysis of *C. officinalis* and three endosymbiotic *Aspergillus* sp. isolated from its inner tissues had led to the identification of 76 secondary metabolites.

## Graphical abstract

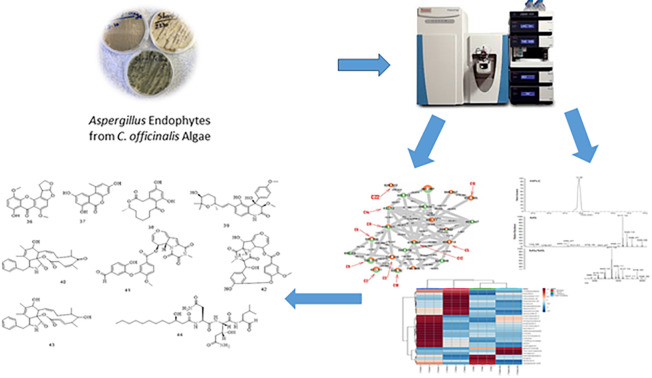

## 1 Introduction

The expanding number of marine-derived natural products described from endosymbiotic fungi encourages additional action and in-depth research for marine environment-derived natural bioactive compounds from a drug discovery perspective.^1,2^ Macroalgae are divided into three primary phyla based on their coloration, which includes red seaweed (*Rhodophyta*), brown seaweed (*Phaeophyta*), and green seaweed (*Chlorophyta*).^3^*Corallina officinalis*, a red seaweed, has a long history of use in traditional Chinese medicine and is a well-known edible seaweed in China and many other nations.^4^

Red algae are known to contain a variety of secondary metabolites, including sulfated sugars, halogenated mono- and diterpenes, sterols, alkaloids, and polyphenols. Many reports demonstrated the biological impact of the secondary metabolites isolated from genus *Corallina* and the endophytes isolated thereof; for example, The *in vitro* assay of total extract of *C. officinalis* exhibited antiprotozoal activity against *Trypanosoma cruzi*.^5^ The new cyclic depsipeptides isolated from culture broth of *Staphylococcus* sp. derived from *C. officinalis*, known as cyclo (2α, 3-diamino-propoincacid-l-Asn-3-β-hydroxy-5-methyl-tetradecanoicacid-l-Leu1-l-Asp-l-Val-l-Leu2-l-Leu3) and cyclo(l-pro-l-omet) exhibiting potent antibacterial and antifungal activities.^6–8^

*Aspergillus* is a large genus with more than 180 different anamorphic species distributed in various ecological niches. As a common fungal endosymbiont, it is regarded as a source of vast classes of chemical compounds with interesting biological functions. Diverse *Aspergillus* species have demonstrated their capacity to produce a wide range of secondary metabolites, including butenolides, alkaloids, terpenoids, cytochalasins, phenalenones, terphenyls, xanthones, sterols, and anthraquinone derivatives with a variety of biological activities, including anti-cancer, anti-fungal, anti-bacterial, anti-viral, anti-inflammatory, anti-trypanosomal and anti-leishmanial activities.^8–14^

Recently, metabolomic studies became an indispensable tool in natural products chemistry where it provides broad qualitative and quantitative profiles of metabolites in organisms under diverse environmental situations. Under stressful circumstances, both plants and microbes create a wide variety of metabolites with distinct chemistries and bioactivities. Through integrated softwares, platforms, libraries and databases it is possible to unveil the intricate interactions between endophytes and their host organisms.^15^

UPLC/MS/MS provides a highly sensitive and adaptable tool and represents the basic tool in metabolomic studies with the ability to analyze minor chemicals providing crucial structural information for identification. However, massive volumes of spectra may be produced using mass spectrometry, which increases the complexity of the analysis. Considering that, the online platform Global Natural Products Social Molecular Networking (GNPS) allows the use of several different mass spectrometry-based metabolomics tools to analyze large sets of data. Moreover, numerous tools offered by GNPS have the ability to automatically search for a spectral match, and the organization provides a public spectrum library.^16,17^

Finding a link between the secondary metabolites produced by the host organism and those produced by the endophytic community is essential to unveil the ecological significance of endophytes and to open new avenue to discover the need of such mutualistic relationship.

In the present study and in continuation of our ongoing research on marine endosymbiotic fungal products, we herein report a comparative LC-MS-MS metabolomics study on the ethyl acetate extracts of the red algae *C. officinalis* and three endosymbiotic fungi isolated from its inner tissue namely; *A. nidulans*, *A. flavipes* and *A*. *flavus*. The identified metabolites belonged to various chemical classes such as alkaloids, anthraquinones, polyketides, sesquiterpenes, butyrolactones, peptides, fatty acids, isocoumarins, quinones, among other miscellaneous compounds.

## 2 Experimental

### 2.1. Fungal material

The fungi *A. nidulans*, *A. flavipes* and *A. flavus* were isolated from the inner tissues of the red algae *C. officinalis*. The algae was collected in the Mediterranean Sea close to Alexanderia, Egypt, in September 2018. For isolation of the fungal strain, the Algae was rinsed with distilled water and then surface sterilization using 70% ethanol was performed for 2 min. Small samples from the inner tissues of the algae were aseptically cut using sterilized blade and pressed onto malt agar plate (15 g per L malt extract, 15 g per L agar, 0.2 g per L chloramphenicol to suppress bacterial growth, pH adjusted to 7.4–7.8 using 10% NaOH). After incubation at 25 °C the fungal strains under investigation were found to grow out of the algal tissue. Pure fungal strains were grown by repeated reinoculation on fresh culture media.

### 2.2. Identification of the fungal strains

The isolated fungal strains were identified as *A. nidulans*, *A. flavipes* and *A. flavus* using a molecular biological protocol by DNA amplification and sequencing of the ITS region as previously reported.^18^ The obtained data of sequencing were submitted to GenBank with the accession number OQ930448 for *A. nidulans*, OQ930542 for *A. flavipes* and OR120990 *for A*. *flavus*.

### 2.3. Cultivation and extraction

Small scale fermentation of the fungal strains was performed on solid rice culture media (100 g rice in 110 mL distilled water, autoclaved for 20 min at 121 °C) in 1 L Erlenmeyer flask (3 flasks) for 30 days at 25 °C under static conditions. After incubation, the fungal cultures were extracted with ethyl acetate, filtered, and evaporated with a rotary evaporator to yield the ethyl acetate extracts for *A. nidulans* (350 mg), *A. flavipes* (400 mg) and *A. flavus* (500 mg). Similarly, the algae for *C. officinalis* was extracted and evaporated to yield 400 mg total extract. Aliquots (10 mg) of each extraction was dissolved in 1 ml of 50% methanol per water, centrifuged at 14 000 rpm for 5 min and filtered through nylon syringe filters (0.22 μm) before subjected to further analysis.

### 2.4. Ultra-performance liquid chromatography (UPLC) analysis

The metabolites of the extracts were analyzed on a reversed phase C18 column (High Strength Silica (HSS) T3, 100 mm × 2.1 mm, 1.7 μm diameter particles, Waters™, Waters Corporation, Milford, MA 01757, USA), connected to ultra-performance liquid chromatography (LC) system (Waters™ Acquity UPLC system, Waters Corporation, Milford, MA 01757, USA).^16^ The injection volume was four μL and the flow rate was adjusted to 400 μL min^−1^. The mobile phases used for chromatographic separation were water containing 0.1% formic acid (A) and acetonitrile containing 0.1% formic acid (B). The following gradient was applied: 1 min 99% A, 13 min linear gradient from 99% A to 45% A, 14.5 min linear gradient from 45% A to 30% A, 15.5 min linear gradient from 30% A to 1% A. The gradient was hold at 1% A from 15.5 to 17 min, followed by linear gradient from 1% A to 99% A to 17.5 min. Finally, the column was re-equilibrated for 2.5 min at 99% A.

### 2.5. High-resolution electrospray ionisation orbitrap mass spectrometry (HR-ESI-orbitrap-MS) analysis

The mass spectra were acquired, covering a mass range 100–1500 *m*/*z*, by orbitrap-type high resolution MS and MS/MS (Thermo Scientific™ Exactive™, Thermo Fisher, Bremen, Germany).^19^ Collision Induced Dissociation (CID) was obtained using a normalized collision energy of 35 eV. Tandem mass spectrometry (MS/MS) data were acquired by using the data-Independent acquisition in both in negative and positive ion modes. Instrument control, data acquisition and processing were performed using Xcalibur software package (Thermo Fischer Scientific, San Jose, CA, USA).

### 2.6. LC-MS-based data processing and multivariate statistical analysis

LC-MS/MS data processing was performed using Mass Spectrometry-Data Independent Analysis (MS-DIAL) software.^20^ The following parameters were used: MS and MS/MS tolerance of 0.01 and 0.05 Da, respectively, retention time = 2–17 min, MS mass range = 50–1500 Da, minimum peak height = 1 × 10^3^ amplitude and retention time tolerance of 0.25 min. Post MS-DIAL data processing, the GNPS export files were imported into the GNPS platform using the WinSCP server.^21^ The GNPS feature processing was achieved following specific parameters such as a fragment ion mass tolerance of *m*/*z* (0.25 Da), a minimum number of common fragment ions (5), and a minimum cosine score (0.7). Subsequently, a search of the bronze spectral library was conducted, with the top 10 hits per spectrum. Metabolomic data analysis and interpretation were performed using MetaboAnalyst.^22^ The resulting data matrix (.csv file) was directly imported to the Metaboanalyst 5 platform (https://www.metaboanalyst.ca/). The dataset was then pareto-scaled and log_2_ transformed to standardize variables and minimize redundancy. Subsequently, the data was subjected to different statistical analysis methods, including principal component analysis (PCA) and hierarchical cluster analysis (HCA), as unsupervised methods, next to Partial Least Squares Discriminant Analysis (PLS-DA) as a supervised method.

## 3 Results and discussion

### 3.1. Chemical profiling of the red alga *C. officinalis*, and associated *Aspergillus* sp.

**Fig. 1:**
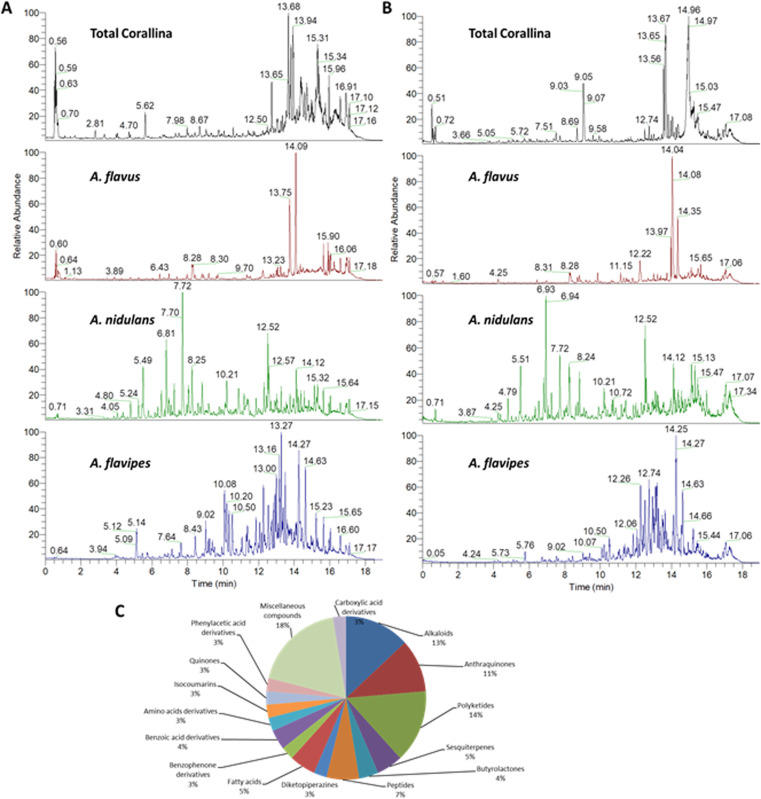
Chemical profiling of the red alga *C. officinalis*, and endosymbiotic *Aspergillus* fungi extracts. Total ion chromatograms (TIC) of metabolites measured by UPLC-MS-MS in negative (A) and positive (B) ionization modes. Percentages of the different classes of annotated metabolites (C).

The chemical profiles of the ethyl acetate extracts from the red alga *C. officinalis*, and associated *Aspergillus*. species (*A. flavipes*, *A. flavus* and *A. nidulans*) were analyzed by LC-ESI-HRMS analysis which was achieved using alternating full scan (FS) and all ion fragmentation (AIF) modes in positive (+) and negative (−) modes. Representative chromatograms are shown in Fig. 1. In total 76 compounds were annotated based on retention times, accurate mass, fragmentation pattern using the available literature^23–25^ as well as the MS/MS databases and bioinformatics analysis using GNPS^21^ (Tables 1 and S1†). The annotated compounds included 11 polyketides, 8 anthraquinones, 12 alkaloids, 5 peptides, 4 sesquiterpenes, 3 butrylactone derivatives, 2 benzophenone derivatives, 4 fatty acids, 2 quinones, 3 amino acids, 7 carboxylic acids and 15 miscellaneous compounds (Fig. 1).

**Table 1:** Annotated metabolites in the ethyl acetate extracts of the red alga, *C. officinalis*, and associated endosymbiotic fungi namely, *A. flavus*, *A. nidulans*, and *A. flavipes* using UPLC-MS/MS analysis in both positive and negative modes

Peak no.	*t*_R_ (min)	Annotated compound	Class	Relative abundance^a^				Ref.
				Total *Corallina*	*A. flavus*	*A. nidulans*	*A. flavipes*	
1	4.06	Orsellinic acid	Benzoic acid derivatives	+++	++	+++	+++	9
2	4.14	*p*-Hydroxyphenyl acetic acid	Phenylacetic acid derivatives	++	++	++	++	^45^kra
3	4.17	5-Hydroxyaverantin	Anthraquinones	−	++	++	+	GNPS
4	5.09	Terrein	Polyketides	++	++	++	+++	GNPS
5	5.13	3-Methylorsellinicacid	Benzoic acid derivatives	++	+	++	++	46
6	5.20	Kojic acid methyl ether	Gamma-pyrone derivatives	++	++	++	+++	47
7	5.33	Insulicolide A	Sesquiterpenes	+	−	−	++	48
8	5.45	Orsellinaldehyde	Dihydroxybenzaldehydes	++	++	++	+++	49
9	5.63	Sinapine	Alkaloids	++	+++	++	++	GNPS
10	6.18	*N*-acetyl-l-leucine	Amino acids	+++	+++	+++	+++	GNPS
11	6.43	Penidiamide	Peptides	+	+++	++	+	GNPS
12	6.60	Asperflavin	Anthraquinones	++	+	+	++	50
13	6.78	Phenylalanine	Amino acids	++	+++	++	++	GNPS
14	6.92	Speradine D	Alkaloids	++	++	+++	−	51
15	6.92	Cichorine	Alkaloids	++	++	++++	++	52
16	7.71	Ferulic acid	Cinnamic acid derivatives	++	++	++++	+++	53
17	7.71	Nidulol	Furane derivatives	+	++	++++	++	54
18	7.97	2-*O*-methyl-9-dehydroxyeurotinone	Anthraquinones	+	++	++	+++	55
19	8.36	Asperfuranone	Polyketides	++	++	++	++	56
20	8.40	4-Hydroxyphenylpyruvic acid	Phenylpyruvic acid derivatives	+++	++	++	+++	GNPS
21	8.66	Homogenentisic acid	Phenylacetic acid derivatives	+	++	++	++	GNPS
22	8.69	Alternariol monomethyl ether	Isocoumarins	−	+	+++	++	57
23	8.96	Daidzein	Isoflavonoids	+++	++	+	+	GNPS
24	9.02	Sterigmatocystin hemiacetal	Xanthone polyketides	+	++	++	++++	58
25	9.20	Erythroglaucin	Quinones	−	+	+++	+	59
26	9.22	1-Hydroxy-6-methyl-8-hydroxymethylxanthone	Xanthone polyketides	+	−	+++	+	GNPS
27	9.32	2,3,6,8,9-Pentahydroxy-1-oxo-3-(2-oxopropyl)-1,2,3,4-tetra-hydroanthracene-2-carboxylic acid	Carboxylic acid derivatives	−	++	++	+++	GNPS
28	9.39	Sterigmatocystin	Xanthone polyketides	+	++	+	++++	58, 60 and 61
29	9.45	Atrochryson carboxylic acid	Carboxylic acid derivatives	+	++	++	++++	GNPS
30	9.70	Aspergilloid C	Sesquiterpenes	++	+++	++	+	62
31	9.77	Violaceol I	Diphenyl- ethers	+	+	++++	++	GNPS
32	9.80	Versiquinazoline E	Alkaloids	++	−	++	+	63
33	9.93	Aspulvinone E	Butenolides	+	+	++	+++	GNPS
34	10.02	Aspergiterpenoid A	Sesquiterpenes	++	++	++	++	64
35	10.08	Epicoccolide B	Polyketides	−	++	++	++++	65
36	10.20	5-Methoxydihydrosterigmatocystin	Xanthone polyketides	++	++	++	++++	58
37	10.87	Alternariol	Isocoumarinss	−	++	++++	++	57
38	11.33	Curvularin	Polyketide	++	+++	+++	++++	GNPS
39	11.96	Aspoquinoline C	Alkaloids	+	+	+	++	66
40	12.12	Cytochalasin Z17	Alkaloids	+	+	+	+++	67
41	12.35	Dethiosecoemestrin	Diketopiperazines	−	−	+++	−	GNPS
42	12.58	Emestrin	Alkaloids	−	+	+++	−	68
43	12.67	Cytochalasin z8	Alkaloids	+	+	+	+++	67
44	12.70	Fellutamide A	Peptides	+	−	++	+++	69
45	12.74	Acyl-hemiacetal sterigmatocystin	Xanthone polyketides	−	+	+++	−	58
46	12.80	Butyrolactone I	Butyrolactone derivatives	++	++	+++	++++	GNPS
47	13.27	Butyrolactone VII	Butyrolactone derivatives	+	++	++	++++	GNPS
48	13.27	2-*O*-methylbutyrolactone II	Butyrolactone derivatives	−	+	+	+++	GNPS
49	13.32	Aspergillide E	Macrolides	+	+	+++	++	70
50	13.40	Emericellamide C	Peptides	+	+	+++	−	71
51	13.45	Fellutamide D	Peptides	+	++	++	+++	72
52	13.49	Emodin	Anthraquinones	++	++	+++	++	GNPS
53	13.49	Eurotinone	Anthraquinoness	−	++	++	++++	73
54	13.49	Monodictyphenone	Polyketides	−	++	++	++++	74
55	13.72	Cyclopiazonic acid	Alkaloids	+	+++	+++	+	75
56	13.76	Sydonic acid	Sesquiterpenes	++++	++++	++++	++++	76
57	13.92	Emericellamide A	Polyketides	++	+	+++	−	71
58	13.99	Asperphenamate	Phenylalanine derivatives	+	++	++	+++	77
59	13.99	2-(((2-Ethylhexyl)oxy)carbonyl)benzoic acid	Benzoic acid derivatives	+++	+++	++	+++	GNPS
60	14.08	2-Methyleurotinone	Anthraquinones	++	++	++	++++	78
61	14.11	Emericellamide E	Polyketides	+++	−	++++	++	71
62	14.15	1-Hexadecanoyl glycerophosphocholine	Lipids	+++	+++	++	++	GNPS
63	14.22	8-Hydroxy-9,12-octadecadienoic acid	Fatty acids	+++	+++	+++	+++	GNPS
64	14.25	6,8-*O*-dimethylaverantin	Anthraquinones	+	++	++	++++	GNPS
65	14.25	5,2′-Dihydroxy-3,7,8-trimethoxyflavone	Flavonoids	−	−	+	+++	GNPS
66	14.26	Hormonemate F	Polyketides	++	++	++	++++	GNPS
67	14.35	Flavoglaucin	Quinones	+++	++	++	+	59
68	14.37	Scopularide E	Peptides	++	+	+++	−	GNPS
69	14.56	Aversin	Anthraquinones	−	++	++++	++	61
70	14.5	7-Hydroxy-8,14-dimethyl-9-hexadecanoic acid	Fatty acids	+++	+++	+++	+++	79
71	14.69	Isoechinulins C	Diketopiperazines	+	++	++	++	80
72	14.91	Wentinoid A	Diterpenoids	++	++	++	++	81
73	15.62	Glycerol linoleate	Fatty acids	+++	+++	+++	+++	47
74	15.65	Arugosin G	Benzophenone derivatives	+	+	+	+++	82
75	15.6	Linoleic acid	Fatty acids	++	++	++	++	47
76	16.22	Versiquinazoline (J)	Alkaloids	++	+++	++	++	63

a Symbols represent the relative abundance, (++++) for area ≥1 × 10^9^, (+++) for area ≥1 × 10^7^, (++) for area ≥1 × 10^5^, (+) for area ≥1 × 10^3^, (−) for not detected.

**Fig. 2:**
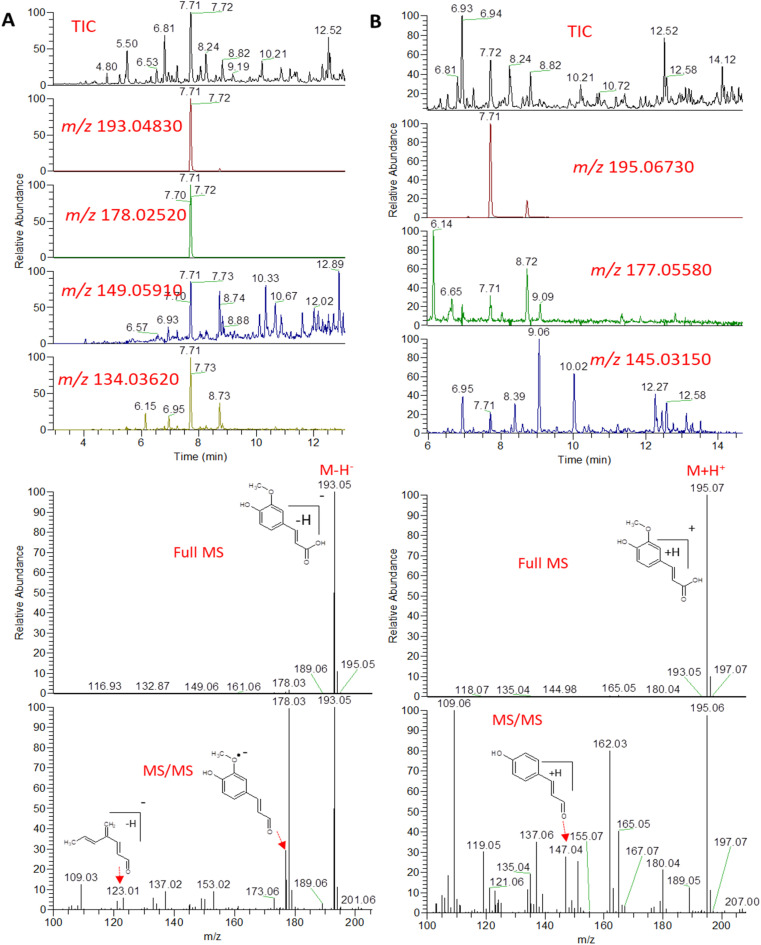
Total ion chromatogram (TIC) and extracted ion chromatograms (EIC) of the peak representing ferulic acid measured by UPLC-MS-MS in negative (A) and positive (B) ionization mode from *A. nidulans* extract.

An example description of the workflow used for annotation of metabolites is described here showing ferulic acid. The identification of ferulic acid was achieved in both negative and positive electrospray ionization modes by the MS^2^ analysis (Fig. 2). In negative ionization mode, a molecular ion peak was observed at *m*/*z* 193.04830 equivalent to the deprotonated adduct [M − H]^−^ with the chemical formula C_10_H_9_O_4_^−^. Characteristic product ion fragments were observed at *m*/*z* 178.02520 (loss of CH_3_ from the precursor ion), *m*/*z* 176.0450 (loss of OH from the precursor ion), *m*/*z* 149.05910 (loss of CO_2_ from the precursor ion) and *m*/*z* 134.03620 (loss of CH_3_ with CO_2_ from the precursor ion).^26,27^ In positive ionization mode, a molecular ion peak was observed at *m*/*z* 195.06730 equivalent to the protonated adduct [M + H]^+^with the chemical formula C_10_H_11_O_4_^+^. Characteristic product ion fragments were observed at *m*/*z* 180.04380 (loss of CH_3_ from the precursor ion), *m*/*z* 177.05580 (loss of water from the precursor ion), [M + H–H_2_O]^+^, and *m*/*z* 147.04610 (loss of OCH_3_ and OH from the precursor ion).^28^ Other example description for the annotated metabolites are described in the detailed classification in the following sections.

#### 3.1.1. Polyketides

Polyketides include a diverse array of natural products with different structural skeletons, however, they all share in common their origin from the assembly of acetate/malonate units over PKS (polyketide synthase enzyme complex); anthraquinone and polyketide alkaloids are subclasses of polyketides characterized by a tricyclic anthraquinone core structure as well as nitrogen containing compounds, respectively.^29^ Polyketides represented the major class of metabolites identified in the studied algae and their three endophytes with a total number of 11 compounds identified in the three species. Emericellamide A (57) is a polyketide identified in positive ionization mode and showed [M + H]^+^molecular ion peak at *m*/*z* 610.41785 with chemical formula C_31_H_56_N_5_O_7_^+^. Emericellamide A fragmentation mechanism in the positive ion mode starts by loss of fragment ion [M + H]^+^*m*/*z* 71.04 with the chemical formula C_3_H_5_NO˙ and the remained moiety was 
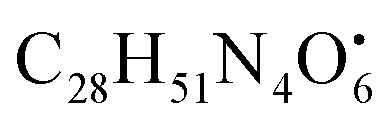
 with [M + H]^+^*m*/*z* 539.3798 followed by continuous cleavage of different amide bonds and loss of different amino acid fragments occurred to this moiety till it reaches the last fragment.^30^ Polyketides were the major secondary metabolites exhibited by the data extracted from LC-MS, where, terrein (4), epicoccolide B (35), curvularin (38), emericellamide A (57), emericellamide E (61) and hormonemate F (66) were detected at different retention times in both negative and positive ionization modes. Compounds 4 was identified as terrain,^24^ it showed [M − H]^−^ at *m*/*z* 153.05495 with characteristic fragments through the loss of C_2_H_6_ unit ([M − H]^−^–30)123.0442 and loss of H_2_O ([M − H]^−^–18)135.0442. Polyketides were the major secondary metabolites exhibited by the data extracted from LC-MS, with total number of 11 where, 6 of which were found in the host algal sample and its three endosymbiotic fungi such as terrein (4) (*t*_R_ 5.09 min), sterigmatocystin hemiacetal (24) (*t*_R_ 9.02 min), sterigmatocystin (28) (*t*_R_ 9.39 min), 5-methoxydihydrosterigmatocystin (36) (*t*_R_ 10.20 min), curvularin (38) (*t*_R_ 11.33 min) and hormonemate F (66) (*t*_R_ 14.26 min). Epicoccolide B (35) (*t*_R_ 10.08 min), was detected in all three *Aspergillus* extracts and totally absent in the host algal extract. Emericellamide E (61) (*t*_R_ 14.11 min) and 1-hydroxy-6-methyl-8-hydroxymethylxanthone (26) (*t*_R_ 9.22 min), were not detected in *Aspergillus flavus* extract, while they were detected in the other three extracts. Acyl-hemiacetal sterigmatocystin (45) (*t*_R_ 12.74 min), was not identified in the host algal extract and its endosymbiotic *A. flavipes*, while was detected in *A. flavus* and *A. nidulans* extracts. Emericellamide A (57) (*t*_R_ 13.92 min), was not detected in *A. flavipes* extract, while was detected in the remaining three sample extracts.

#### 3.1.2. Anthraquinones

Anthraquinones are a subclass of polyketide natural products demonstrating a wide range of biological activities and possible industrial application including cytotoxic, antiplasmodial, anticancer, antitumor, algaecide, antifungal, enzyme-inhibiting, antiplatelet aggregation, antibacterial, antiviral, and phytotoxic properties.^31,32^ HR-LC-MS analysis revealed the predominance of anthraquinones in the algal extracts and the three endosymbiotic fungi. 2-Methyleurotinone (60), is an anthraquinone compound that showed [M − H]^−^ peak at *m*/*z* 301.0718, 286.0485, 271.0252, and 243.0300, respectively in negative ionization mode. 2-Methyleurotinone was fragmented in MS^2^ analysis to yield product ion at *m*/*z* 286.0485 by loss of OH, *m*/*z* 271.0252 by loss of OCH_3_, and *m*/*z* 243.0300 by loss of CO_2_ and CH_3_ from the precursor ion. A total of 8 anthraquinone derivatives were detected, 5 of which were found in the host algal sample and its three endosymbiotic fungi such as asperflavin (12) (*t*_R_ 6.60 min), 2-*O*-methyl-9-dehydroxyeurotinone (18) (*t*_R_ 7.97 min), emodin (52) (*t*_R_ 13.49 min) (Fig. S1†), 2-methyleurotinone (60) (*t*_R_ 14.08) and 6,8-*O*-dimethylaverantin (64) (*t*_R_ 14.25 min). Also, the other three metabolites, 5-hydroxyaverantin (3) (*t*_R_ 4.17 min), eurotinone (53) (*t*_R_ 13.49 min) and aversin (69) (*t*_R_ 14.56 min) were detected in the three endosymbiotic fungi and totally absent in the host algal sample.

#### 3.1.3. Alkaloids

Alkaloids demonstrate distinctive structural skeletons derived from different amino acids. These nitrogen containing compounds are among the most effective compounds, and many of them had been developed into market drug product, still many others under different phases of clinical trials.^33,34^ Sinapine (9) is an alkaloid that is more stable in negative ionization mode therefore the identification was conducted in positive mode to verify ion breakdown. In positive mode of ions, the molecular ion peak [M]^+^appeared at *m*/*z* 310.16489 (Fig. S2†). Sinapine was fragmented to yield product ion at *m*/*z* 251.0912 by loss of trimethyl amine moiety, then loss of ethoxy group to produce *m*/*z* 207.0651, followed by loss of two methoxy groups to produce *m*/*z* 147.0421, and then loss of hydroxyl group to produce *m*/*z* 131.9743 from the precursor ion.^35^ A total of 12 alkaloids were detected, 8 of which were found in the host algal sample and the three endosymbiotic fungi (*A. flavus*, *A. nidulans* and *A. flavipes*), such as sinapine (9) (*t*_R_ 5.63 min), cichorine (15) (*t*_R_ 6.92 min), aspoquinoline C (39) (*t*_R_ 11.96 min), cytochalasin Z17 (40) (*t*_R_ 12.12 min), cytochalasin Z8 (43) (*t*_R_ 12.67 min), cyclopiazonic acid (55) (*t*_R_ 13.72 min) (Fig. S3†) and versiquinazoline J (76) (*t*_R_ 16.22 min). On the other hand, dethiosecoemestrin (41) (*t*_R_ 12.35 min) (Fig. S4†), was only observed in *A. nidulans* extract and not observed in the host algal extract, *A. flavus* and *A. flavipes*. Emestrin (42) (*t*_R_ 12.58 min), was observed in *A. flavus* and *A. nidulans*, whereas, not observed in *A. flavipes* and the host algal extract. Speradine D (14) (*t*_R_ 6.92 min) was only not observed in *A. flavipes*, whereas was observed in *A. flavus*, *A. nidulans* and the host algal extract. Versiquinazoline E (32) (*t*_R_ 9.80 min), was only not observed in *A. flavus*, whereas observed in *A. nidulans*, *A. flavipes* and the host algal extract.

#### 3.1.4. Peptides

Five peptides were identified in the host algal extract and there three endosymbiotic fungi. Penidiamide (11) (*t*_R_ 6.43 min) and fellutamide D (51) (*t*_R_ 13.45 min) were detected in all four extracts. Emericellamide C (50) is a peptide identified in positive ionization mode and showed [M + H]^+^ molecular ion peak at *m*/*z* 596.4021with chemical formula C_30_H_54_N_5_O_7_^+^. Emericellamide C fragmentation mechanism in the positive ion mode starts by loss of fragment ion [M + H]^+^*m*/*z* 71.04 with the chemical formula C_3_H_5_NO˙ and the remained moiety was 
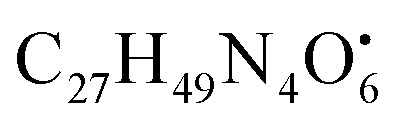
 with [M + H]^+^*m*/*z* 525.3638 witnessed continuous cleavage of different amide bonds and loss of different amino acid fragments to produce different product ions including 454.3265, 341.2424, and 244.1647.^30^ Emericellamide C (50) (*t*_R_ 13.44 min) and scopularide E (68) (*t*_R_ 14.37 min) (Fig. S5†) were not detected only in *A. flavipes* extract, while detected in the other three extracts. Fellutamide A (44) (*t*_R_ 12.70 min), was detected in *A. nidulans* extract, *A. flavipes* extract and the host algal extract, whereas, not detected in *A. flavus* extract.

#### 3.1.5. Fatty acids

LC-MS analysis revealed the predominance of fatty acids and their hydroxides in the host algal extract and their all endosymbiotic fungal extracts. A total of 4 fatty acids were detected. 8-Hydroxy-9, 12-octadecadienoic acid (63) (*t*_R_ 14.22 min), 7-hydroxy-8, 14-dimethyl-9-hexadecanoic acid (70) (*t*_R_ 14.5 min), glycerol linoleate (73) (*t*_R_ 15.62 min) and linoleic acid (75) (*t*_R_ 15.6 min), were observed in all extracts.

#### 3.1.6. Amino acids and organic acid derivatives

Three amino acids, two carboxylic acids, three benzoic acids and two phenyl acetic acid derivatives were identified in the examined algae and their endosymbiotic fungal extracts. *N*-Acetyl-l-leucine (10) is an amino acid derivative showed molecular ion [M + H]^−^ peak at *m*/*z* 172.09734 with the chemical formula C_8_H_14_NO_3_ in negative electrospray ionization mode. Characteristic product ion fragment was observed at *m*/*z* 130.0866 (loss of acetyl group CH_3_CO after cleavage of amide bond from the precursor ion).^36^ Asperphenamate (58) (phenylalanine derivative) showed molecular ion [M + H]^+^peak at *m*/*z* 507.22897 with the chemical formula C_32_H_31_N_2_O_4_^+^ in the positive electrospray ionization mode. Characteristic product ion fragments were observed at *m*/*z* 256.1317 (loss of 
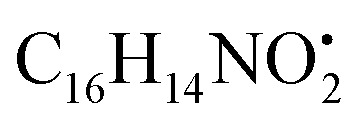
 from the precursor ion), and *m*/*z* 238.1213 (loss of 
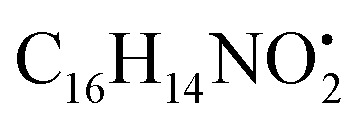
 from the precursor ion).^37^ The identified amino acids, *N*-acetyl-l-leucine (10), (*t*_R_ 6.18 min), phenylalanine (13), (*t*_R_ 6.78 min) and asperphenamate (58) (*t*_R_ 13.99 min) were observed in *A. flavus*, *A. nidulans*, *A. flavipes* and the algal extracts. Two identified carboxylic acid derivatives, 2, 3, 6, 8, 9-pentahydroxy-1-oxo-3-(2-oxopropyl)-1,2,3,4-tetra-hydroanthracene-2-carboxylic acid (27), (*t*_R_ 9.32 min), was only not observed in the host algal extract. On the other hand, atrochryson carboxylic acid (29) (*t*_R_ 9.45 min), was observed in all extracts. All three identified benzoic acid derivatives were observed in host algal extract and their three endosymbiotic fungal extracts, such as orsellinic acid (1) (*t*_R_ 4.06 min), 3-methylorsellinic acid (5) (*t*_R_ 5.13 min) and 2-(((2-ethylhexyl)oxy)carbonyl)benzoic acid (59) (*t*_R_ 13.99 min) (Fig. S6†). Orsellinic acid (1) (benzoic acid derivative) showed molecular ion [M − H]^−^ peak at *m*/*z* 167.03418 with the chemical formula C_8_H_7_O_4_^−^ in negative electrospray ionization mode. Characteristic product ion fragments were observed at *m*/*z* 151.0393 (loss of OH from the precursor ion), and *m*/*z* 123.0443 (loss of carboxylic group from the precursor ion).^38,39^ Also two identified phenyl acetic acid derivatives were observed in all examined extracts, including *p*-hydroxyphenyl acetic acid (2) (*t*_R_ 4.14 min) and homogenentisic acid (21) (*t*_R_ 8.66 min).

#### 3.1.7. Butrylactone derivatives

Butyrolactone VII (47) (benzoic acid derivative) showed molecular ion [M + H]^+^peak at *m*/*z* 439.1755 with the chemical formula C_25_H_27_O_7_^+^ in the positive electrospray ionization mode. Characteristic product ion fragments were observed at *m*/*z* 422.2278 (loss of OH from the precursor ion), *m*/*z* 331.1309 (loss of two OH groups and 
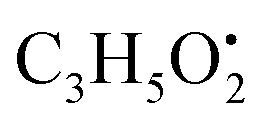
 from the precursor ion), and *m*/*z* 175.1107 (loss of 
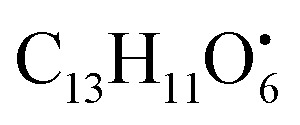
 from the precursor ion)^40^ (Fig. S7†). Three butrylactone derivatives were identified in algal extract and there endosymbiotic fungal extracts. Butyrolactone I (46) (*t*_R_ 12.80 min) (Fig. S8†) and butyrolactone VII (47) (*t*_R_ 13.27) were observed in *A. flavus*, *A. nidulans*, *A. flavipes* and algal extracts. 2-*O*-methylbutyrolactone II (48) (*t*_R_ 13.27 min) was totally not observed in the host algal extract, whereas observed in all three endosymbiotic fungal extracts (Fig. S9†).

#### 3.1.8. Benzophenone, sesquiterpene and quinone derivatives

Monodictyphenone (54) (*t*_R_ 13.49 min), was only not detected in the host algal sample extract, while detected in the remaining extracts. On the other hand, arugosin G (74) (*t*_R_ 15.65 min), was detected in all sample extracts.

Four sesquiterpenes were identified, three of them including aspergilloid C (30) (*t*_R_ 9.70 min), aspergiterpenoid A (34) (*t*_R_ 10.02 min) and sydonic acid (56) (*t*_R_ 13.76 min) were detected in *A. flavus*, *A. nidulans*, *A*. *flavipes* and the algal extracts. On the other hand, insulicolide A (7) (*t*_R_ 5.33 min) was not detected in *A. flavus* and *A. nidulans* extracts, while detected in *A. flavipes* and algal extracts.

Two quinone derivatives were identified. Erythroglaucin (25) (*t*_R_ 9.20 min), was only not detected in the host algal extract, whereas detected in *A. flavus*, *A. nidulans* and *A. flavipes* extracts. On the other hand, flavoglaucin (67) (*t*_R_ 14.35 min) was detected in all four sample extracts.

#### 3.1.9. Miscellaneous compounds

**Fig. 3:**
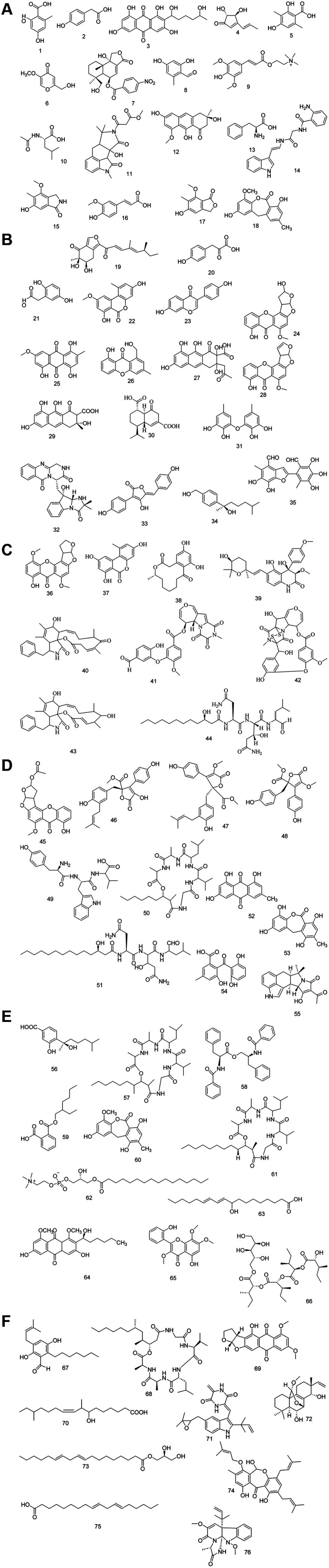
(A) Chemical structures of compounds 1–20. (B) Chemical structures of compounds 21–35. (C) Chemical structures of compounds 36–44. (D) Chemical structures of compounds 45–55. (E) Chemical structures of compounds 56–66. (F) Chemical structures of compounds 67–76.

Alternariol monomethyl ether (22) is an isocoumarin that showed molecular ion [M − H]^−^ peak at *m*/*z* 271.0612 with the chemical formula C_15_H_11_O_5_^−^ in negative electrospray ionization mode. The main product ion fragment was observed at *m*/*z* 228.0429 (loss of CO_2_ from the precursor ion).^41^ Two compounds belong to isocoumarin derivatives, Alternariol monomethyl ether (22) (*t*_R_ 8.69 min) and alternariol (37) (*t*_R_ 10.87), were totally not detected in the host algal sample extract, while were detected in *A. flavus*, *A. nidulans* and *A. flavipes* extracts. The flavonoid, 5,2′-dihydroxy-3,7,8-trimethoxyflavone (65) (*t*_R_ 14.25 min) (Fig. S10†), was only detected in both *A. nidulans* and *A. flavipes* extracts, while this metabolite was totally not detected in *A. flavus* and the algal extracts. On the other hand, the rest of different categories of identified miscellaneous metabolites were detected in all examined sample extracts such as, kojic acid methyl ether (6) (*t*_R_ 5.20 min) belongs to gamma-pyrone derivatives, orsellinaldehyde (8) (*t*_R_ 5.45 min), which belongs to dihydroxybenzaldehyde derivatives, ferulic acid (16) (*t*_R_ 7.71 min) which belongs to cinnamic acid derivatives, asperfuranone (19) (*t*_R_ 8.36 min) which belongs to benzofurane derivatives, 4-hydroxyphenylpyruvic acid (20) (*t*_R_ 8.40 min) which belongs to phenyl acetic acid derivatives, isoflavonoid diadzein (23) (*t*_R_ 8.96 min), diphenyl ether violaceol-I (31) (*t*_R_ 9.77 min), aspulvinone E (33) (*t*_R_ 9.93 min) which belongs to butenolide derivatives (Fig. S11†), macrolide aspergillide E (49) (*t*_R_ 13.32 min), furan derivatives nidulol (17) (*t*_R_ 7.71 min), lipid metabolites 1-hexadecanoyl glycerophosphocholine (62) (*t*_R_ 14.15 min) and diterpenoid wentinoid A (72) (*t*_R_ 14.91 min). Nidulol (17) is a furan derivative identified in positive ionization mode and showed [M + H]^+^ molecular ion peak at *m*/*z* 195.0651with chemical formula C_10_H_11_O_4_^−^. Characteristic product ion fragments were observed at *m*/*z* 180.0412 (loss of CH_3_ from the precursor ion), *m*/*z* 151.0386 (loss of CO_2_ group from the precursor ion), and *m*/*z* 147.0437 (loss of methoxy and OH groups from the precursor ion). Violaceol I (31) is a diphenyl ether with molecular ion [M + H]^−^ peak at *m*/*z* 261.0773 and chemical formula of C_14_H_13_O_5_^−^ in negative electrospray ionization mode. The cleavage in C–O–C bond linked two benzene rings producing two product ions at *m*/*z* 139.0393 and 123.0443 with chemical formula 
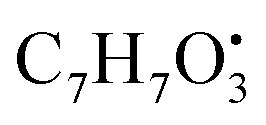
 and 
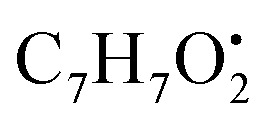
, respectively. Then the loss of CH_3_ from 
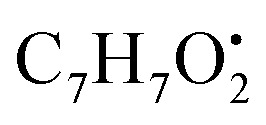
 produced product ion at *m*/*z* 109.0343 with chemical formula 
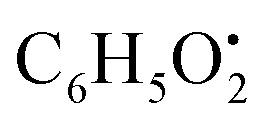
 (Fig. S12†). Representative chemical structures of the annotated metabolites are shown in Fig. 3.

The comprehensive metabolomic study of the three different *Aspergillus* sp. revealed that polyketides, anthraquinoes and alkaloids are the major classes of the identified secondary metabolites. These results comply with the reported data in multiple reviews comprehensively describing hundreds of compounds belonging to the three mentioned classes of secondary metabolites from marine derived *Aspergillus* sp.^42–44^ Moreover, the presence of common compounds in the host algae *C. officinalis* as well as the three isolated *Asprgillus* endosymbionts unveils the presence mutualistic symbiotic relationship emphasizing the ecological significance the fungal endophytes, where they can provide bioactive metabolites as chemical defense strategy to the host organism and in turn secures a nutrient rich system to flourish.

### 3.2. Multivariate analysis of the LC-ESI-HRMS data

**Fig. 4:**
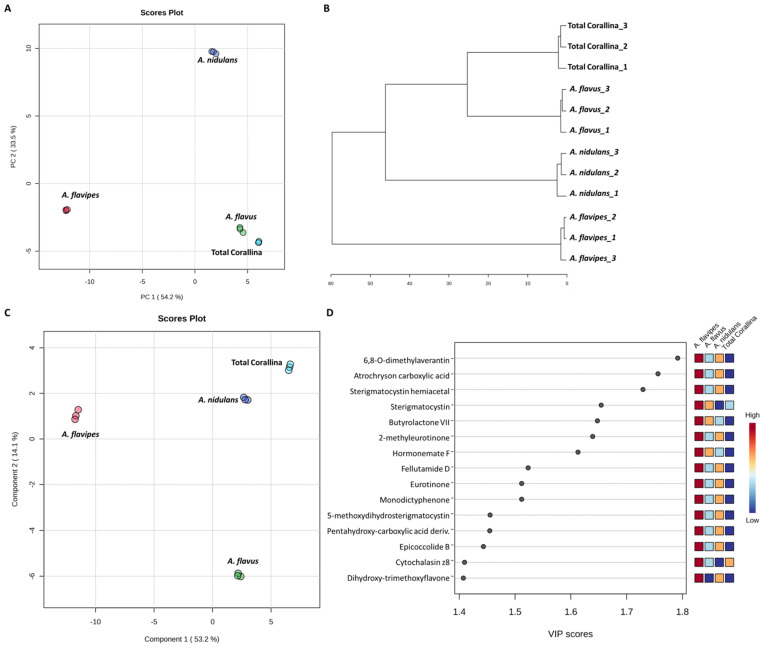
Multivariate statistical analysis of liquid chromatography-high resolution mass spectrometry (LC-HRMS) data of *C. officinalis* and associated endosymbiotic *Aspergillus* fungi. Principal component analysis (PCA) score plot (A), hierarchical cluster analysis (HCA) dendrogram (B), Partial Least Squares Discriminant Analysis (PLS-DA) score plot (C) and variable importance in projection (VIP) scores of the top 15 significant metabolites (D).

**Fig. 5:**
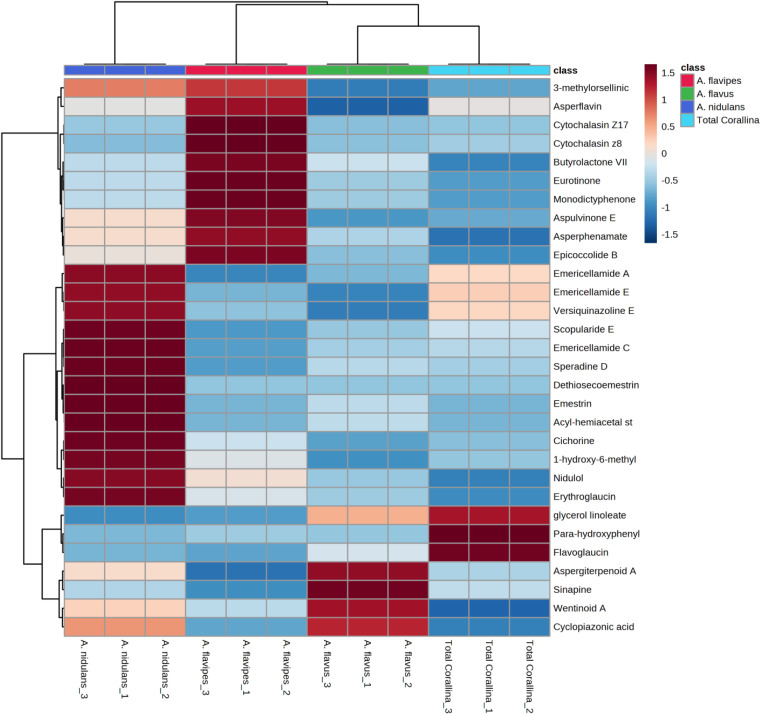
Heatmap of the top 30 metabolites differentially changing between *C. officinalis* and associated endosymbiotic *Aspergillus* fungi based on the results derived from the liquid chromatography-high resolution mass spectrometry (LC-HRMS) data. All metabolites presented here have *p* < 0.05 based on ANOVA results and a fold-change> 2.

Non-targeted metabolomics approaches based on liquid chromatography-high resolution mass spectrometry (LC-HRMS) have been widely used for specific discrimination of different biological samples particularly from plants and fungi.^83,84^ Chemometrics tools such as principal component analysis (PCA), hierarchical clustering analysis (HCA) and partial least squares discriminant analysis (PLS-DA) provide a wealth of techniques for exploratory analysis and classification of multivariate data. The LC-MS-MS data of the total extract as well as the associated fungal extracts were subjected to multivariate analysis to evaluate their chemical diversity. Unsupervised classifications including principal component analysis (PCA) and hierarchical clustering analysis (HCA) were used to simplify a large amount of data without compromising the main information (Fig. 4A and B). PCA analysis revealed clustering of *A. flavipes* from *C. officinalis* and other associated endosymbiotic *Aspergillus* fungi (*A. flavus* and *A. nidulans*) among the first principal component (PC1) which accounted for 54.2% of the variance (Fig. 4A). On the other hand, the second principal component (PC2), which accounted for 33.5% of the variance, revealed a clear separation of *A. nidulans* from *A. flavus* and *Corallina officinalis* which were clustered together. HCA analysis revealed two main clusters; the first cluster included *A. flavipes* while the second cluster revealed separation of *A. nidulans* from *A. flavus* and *C. officinalis*, consistent with PCA results (Fig. 4B). Heatmap of the top 30 metabolites differentially changing between *C. officinalis* and associated endosymbiotic *Aspergillus* fungi is depicted in Fig. 5.

Supervised classifications, on the other hand, including partial least squares discriminant analysis (PLS-DA) was used as multivariate dimensionality-reduction tool for discriminative variable selection (Fig. 4C). PLS-DA achieved the effective discrimination between the tested groups. A Variable Importance for Projection (VIP) score is a measure of a variable importance in the PLS-DA model. It summarizes the quantitative contribution of a variable to the model. The candidate metabolites with variable importance in the PLS-DA (VIP) responsible for the discrimination and with scores ≥1 were considered important in the PLS-DA model (Fig. 4D). Anthraquinones such as eurotinone, 2-methyleurotinone and 6,8-*O*-dimethylaverantin were found to be discriminatory metabolites for *A. flavipes*. Additionally, polyketides such as epicoccolide B, monodictyphenone and hormonemate F as well as other xanthones derivatives such as sterigmatocystin, sterigmatocystin hemiacetal and 5-methoxydihydrosterigmatocystin influence the metabolic discrimination of *A. flavipes* from *Corallina officinalis* and other associated endosymbiotic *Aspergillus* fungi (*A. flavus* and *A. nidulans*). Further, alkaloid derivatives such as sinapine, cyclopiazonic acid and versiquinazoline (J) together with fatty, benzoic and amino acids derivatives (*e.g. N*-acetyl-leucine, phenylalanine, 2-(((2-Ethylhexyl)oxy)carbonyl)benzoic acid, 8-Hydroxy-9,12-octadecadienoic acid, 7-hydroxy-8,14-dimethyl-9-hexadecanoic acid and glycerol linoleate) dominated *A. flavus*. Additionally, penidiamide, curvularin and aspergilloid C were found to discriminate this fungal species. Moreover, metabolites from diverse chemical classes such as cichorine, aversin, violaceol I, nidulol, alternariol and emericellamide E were found to be abundant in *A. nidulans*, discriminating it from *C. officinalis* and other associated endosymbiotic *Aspergillus* fungi. *C. officinalis* was found to accumulate flavoglaucin, emericellamide E, daidzein, orsellinic acid next to some fatty acids and organic acid derivatives.

## 4 Conclusions

In this study, we investigated the metabolic profile of *C. officinalis* and the three isolated endophytes *A. nidulans*, *A. favipes*, and *A. flavus* using LC-MS/MS. *C. officinalis* and its endosymbiotic fungi demonstrate a valuable source of various metabolites including alkaloids, polyketides, sesquiterpenes, butyrolactones, peptides, fatty acids, isocoumarins, and quinones. Based on LC-MS/MS, polyketides represented the highest percentage (14%) among other metabolite classes of *C. officinalis* and related endosymbiotic *Aspergillus* fungi, followed by alkaloids (13%), anthraquinones (11%), peptides (7%), fatty acids and sesquiterpenes (5% for each one), in addition to small amounts of other classes such as butyrolactones, diketopiperazines, isocoumarins, amino acid derivatives, benzoic acid derivatives, and other miscellaneous classes. All metabolites that were found in *C. officinalis* were also exist in its endophytes with different proportions. On the contrary, some metabolites have been identified in endosymbiotic fungi and did not exist in their host. The chemical profiling of many groups of algae and their endophytes is still unrevealed and needs to be explored. These findings will be of interest in pharmaceutical applications for drug discovery from natural resources. Further studies also should be pushed toward investigating the correlations between these endophytes and their hosts while predicting the viable applications and pharmacological effects.

## Author contributions

Sherif M. Shama: methodology, investigation, data curation, and writing – original draft, Ahmed M. Elissawy: writing – original draft, writing – review & editing, and supervision Mohamed A. Salem: methodology, data curation, formal analysis, writing – original draft, writing – review & editing, and supervision, Fadia S. Youssef: writing – review & editing and supervision, Mohamed S. Elnaggar: methodology, Hesham R. El-Seedi: formal analysis, writing – review & editing, Shaden A. M. Khalifa: formal analysis, writing – review & editing, Khaled Briki: writing – review, Dalia Ibrahim Hamdan: writing – review & editing and supervision, Abdel Nasser B. Singab: conceptualization, writing – review & editing, and supervision.

## Conflicts of interest

There are no conflicts to declare.

## Supplementary Material

RA-014-D4RA01055H-s001

## References

[cit1] Rashmi M. , Kushveer J. and Sarma V., Endophytes and Secondary Metabolites, Springer International Publishing, Cham, 2019, pp. 491–526

[cit2] Jimenez C. (2018). ACS Med. Chem. Lett..

[cit3] Sahayaraj K. , presented in part at the National Conference on Conservation and Sustainable Utilization of Marine Resources, Tamil Nadu, India, 2015, pp. 22–23

[cit4] Yang Y., Liu D., Wu J., Chen Y., Wang S. (2011). Int. J. Biol. Macromol..

[cit5] Allmendinger A., Spavieri J., Kaiser M., Casey R., Hingley-Wilson S., Lalvani A., Guiry M., Blunden G., Tasdemir D. (2010). Phytother. Res..

[cit6] Abdelhameed R. F. A., Elhady S. S., Noor A. O., Almasri D. M., Bagalagel A. A., Maatooq G. T., Khedr A. I. M., Yamada K. (2019). Metabolites.

[cit7] Sun R. R., Miao F. P., Zhang J., Wang G., Yin X. L., Ji N. Y. (2013). Magn. Reson. Chem..

[cit8] Singab A. N. B., Mostafa N. M., Elkhawas Y. A., Al-Sayed E., Bishr M. M., Elissawy A. M., Elnaggar M. S., Fawzy I. M., Salama O. M., Tsai Y. H., Chang F. R. (2022). Mar. Drugs.

[cit9] Nguyen M. V., Han J. W., Kim H., Choi G. J. (2022). ACS Omega.

[cit10] Romsdahl J., Wang C. C. C. (2019). Medchemcomm.

[cit11] El-Hawary S. S., Moawad A. S., Bahr H. S., Abdelmohsen U. R., Mohammed R. (2020). RSC Adv..

[cit12] Elnaggar M. S., Elissawy A. M., Youssef F. S., Kicsak M., Kurtan T., Singab A. N. B., Kalscheuer R. (2023). RSC Adv..

[cit13] Amr K., Ibrahim N., Elissawy A. M., Singab A. N. B. (2023). Fungal Biol. Biotechnol..

[cit14] Elissawy A. M., Ebada S. S., Ashour M. L., El-Neketi M., Ebrahim W., Singab A. B. (2019). Phytochem. Lett..

[cit15] Tawfike A. F., Viegelmann C., Edrada-Ebel R. (2013). Methods Mol. Biol..

[cit16] Hou Y., Braun D., Michel C., Klassen J., Adnani N., Wyche T., Bugni T. (2012). Anal. Chem..

[cit17] Tangerina M., Furtado L., Leite V., Bauermeister A., Velasco-Alzate K., Jimenez P., Garrido L., Padilla G., Lopes N., Costa-Lotufo L., Ferreira M. (2020). PLoS One.

[cit18] Kjer J., Debbab A., Aly A. H., Proksch P. (2010). Nat. Protoc..

[cit19] Salem M. A., Salama M. M., Ezzat S. M., Hashem Y. A. (2022). Sci. Rep..

[cit20] Tsugawa H., Cajka T., Kind T., Ma Y., Higgins B., Ikeda K., Kanazawa M., VanderGheynst J., Fiehn O., Arita M. (2015). Nat. Methods.

[cit21] Wang M., Carver J. J., Phelan V. V., Sanchez L. M., Garg N., Peng Y., Nguyen D. D., Watrous J., Kapono C. A., Luzzatto-Knaan T., Porto C., Bouslimani A., Melnik A. V., Meehan M. J., Liu W. T., Crusemann M., Boudreau P. D., Esquenazi E., Sandoval-Calderon M., Kersten R. D., Pace L. A., Quinn R. A., Duncan K. R., Hsu C. C., Floros D. J., Gavilan R. G., Kleigrewe K., Northen T., Dutton R. J., Parrot D., Carlson E. E., Aigle B., Michelsen C. F., Jelsbak L., Sohlenkamp C., Pevzner P., Edlund A., McLean J., Piel J., Murphy B. T., Gerwick L., Liaw C. C., Yang Y. L., Humpf H. U., Maansson M., Keyzers R. A., Sims A. C., Johnson A. R., Sidebottom A. M., Sedio B. E., Klitgaard A., Larson C. B., Boya C., Torres-Mendoza D., Gonzalez D. J., Silva D. B., Marques L. M., Demarque D. P., Pociute E., O’Neill E. C., Briand E., Helfrich E. J. N., Granatosky E. A., Glukhov E., Ryffel F., Houson H., Mohimani H., Kharbush J. J., Zeng Y., Vorholt J. A., Kurita K. L., Charusanti P., McPhail K. L., Nielsen K. F., Vuong L., Elfeki M., Traxler M. F., Engene N., Koyama N., Vining O. B., Baric R., Silva R. R., Mascuch S. J., Tomasi S., Jenkins S., Macherla V., Hoffman T., Agarwal V., Williams P. G., Dai J., Neupane R., Gurr J., Rodriguez A. M. C., Lamsa A., Zhang C., Dorrestein K., Duggan B. M., Almaliti J., Allard P. M., Phapale P., Nothias L. F., Alexandrov T., Litaudon M., Wolfender J. L., Kyle J. E., Metz T. O., Peryea T., Nguyen D. T., VanLeer D., Shinn P., Jadhav A., Muller R., Waters K. M., Shi W., Liu X., Zhang L., Knight R., Jensen P. R., Palsson B. O., Pogliano K., Linington R. G., Gutierrez M., Lopes N. P., Gerwick W. H., Moore B. S., Dorrestein P. C., Bandeira N. (2016). Nat. Biotechnol..

[cit22] Pang Z., Chong J., Zhou G., de Lima Morais D. A., Chang L., Barrette M., Gauthier C., Jacques P. E., Li S., Xia J. (2021). Nucleic Acids Res..

[cit23] Khattab O. M., El-Kersh D. M., Khalifa S. A. M., Yosri N., El-Seedi H. R., Farag M. A. (2023). Plants.

[cit24] El-Din M. I. G., Fahmy N. M., Wu F. L., Salem M. M., Khattab O. M., El-Seedi H. R., Korinek M., Hwang T. L., Osman A. K., El-Shazly M., Fayez S. (2022). Plants.

[cit25] El-Garawani I. M., El-Sabbagh S. M., Abbas N. H., Ahmed H. S., Eissa O. A., Abo-Atya D. M., Khalifa S. A. M., El-Seedi H. R. (2020). Sci. Rep..

[cit26] Hong Y., Wang Z., Barrow C. J., Dunshea F. R., Suleria H. A. R. (2021). Antioxidants.

[cit27] Kramberger K., Barlic-Maganja D., Bandelj D., Baruca Arbeiter A., Peeters K., Miklavcic Visnjevec A., Jenko Praznikar Z. (2020). Metabolites.

[cit28] Seraglio S. K. T., Valese A. C., Daguer H., Bergamo G., Azevedo M. S., Gonzaga L. V., Fett R., Costa A. C. O. (2016). Food Res. Int..

[cit29] Mund N. K., Cellarova E. (2023). Biotechnol. Adv..

[cit30] Nielsen K. F., Larsen T. O. (2015). Front. Microbiol..

[cit31] Masi M., Evidente A. (2020). Toxins.

[cit32] Li H., Liang C., Chen Q., Yang Z. (2011). Med. Hypotheses.

[cit33] Rida P. C., LiVecche D., Ogden A., Zhou J., Aneja R. (2015). Med. Res. Rev..

[cit34] Pirillo A., Catapano A. L. (2015). Atherosclerosis.

[cit35] Oszmiański J., Kolniak-Ostek J., Wojdyło A. (2013). Eur. Food Res. Technol..

[cit36] Cui L., Liu Y., Liu M., Ren M., Ahmed A. F., Kang W. (2022). J. Future Foods.

[cit37] Kildgaard S., Mansson M., Dosen I., Klitgaard A., Frisvad J. C., Larsen T. O., Nielsen K. F. (2014). Mar. Drugs.

[cit38] Musharraf S. G., Kanwal N., Thadhani V. M., Choudhary M. I. (2015). Anal. Methods UK.

[cit39] Jin Y., Ma Y., Xie W., Hou L., Xu H., Zhang K., Zhang L., Du Y. (2018). RSC Adv..

[cit40] Wu L., Xie C. L., Yang X. W., Chen G. (2021). Mar. Drugs.

[cit41] Lau B. P., Scott P. M., Lewis D. A., Kanhere S. R., Cleroux C., Roscoe V. A. (2003). J. Chromatogr. A.

[cit42] youssef F., Alshammari E., Ashour M. (2021). Int. J. Mol. Sci..

[cit43] Ghoran S., Taktaz F., Ayatollahi S., Kijjoa A. (2022). Mar. Drugs.

[cit44] Bai X., Sheng Y., Tang Z., Pan J., Wang S., Tang B., Zhou T., Shi L., Zhang H. (2023). J. Fungi.

[cit45] Zuo W. J., Jin P. F., Dong W. H., Dai H. F., Mei W. L. (2014). Chin. J. Nat. Med..

[cit46] Chen G. D., Hu D., Huang M. J., Tang J., Wang X. X., Zou J., Xie J., Zhang W. G., Guo L. D., Yao X. S., Abe I., Gao H. (2020). Chem. Commun..

[cit47] Eliwa E. M., El-Metwally M. M., Halawa A. H., El-Agrody A. M., Bedair A. H., Shaaban M. (2017). J. At. Mol..

[cit48] Rahbaek L., Christophersen C., Frisvad J., Bengaard H. S., Larsen S., Rassing B. R. (1997). J. Nat. Prod..

[cit49] Han H., Yu C., Qi J., Wang P., Zhao P., Gong W., Xie C., Xia X., Liu C. (2023). Microb. Cell Fact..

[cit50] Yang X., Kang M. C., Li Y., Kim E. A., Kang S. M., Jeon Y. J. (2017). Molecules.

[cit51] Uka V., Moore G. G., Arroyo-Manzanares N., Nebija D., De Saeger S., Diana Di Mavungu J. (2017). Toxins.

[cit52] Sanchez J. F., Entwistle R., Corcoran D., Oakley B. R., Wang C. C. (2012). Medchemcomm.

[cit53] Abonyi D. O., Eze P. M., Abba C. C., Ujam N. T., Proksch P., Okoye F. B., Esimone C. O. (2018). Eur. J. Biol. Res..

[cit54] Aukamp P., Holzapfel C. (1968). S. Afr. J. Chem..

[cit55] Yan H. J., Li X. M., Li C. S., Wang B. G. (2012). Helv. Chim. Acta.

[cit56] Wang C. C., Chiang Y. M., Praseuth M. B., Kuo P. L., Liang H. L., Hsu Y. L. (2010). Basic Clin. Pharmacol. Toxicol..

[cit57] Kong K., Huang Z., Shi S., Pan W., Zhang Y. (2023). BMC Microbiol..

[cit58] Song F., Ren B., Chen C., Yu K., Liu X., Zhang Y., Yang N., He H., Liu X., Dai H., Zhang L. (2014). Appl. Microbiol. Biotechnol..

[cit59] Volkov V. V., Perry C. C. (2021). Dyes Pigm..

[cit60] Zhang S. S., Zhu A. O., Bai X., Zhu H. J., Cao F. (2020). Chem. Nat. Compd..

[cit61] Cao T. Q., Liu Z., Dong L., Lee H., Ko W., Vinh L. B., Tuan N. Q., Kim Y. C., Sohn J. H., Yim J. H., Lee D. S., Oh H. (2022). Molecules.

[cit62] Liu Z., Zhao J. Y., Sun S. F., Li Y., Qu J., Liu H. T., Liu Y. B. (2019). J. Nat. Prod..

[cit63] Cheng Z., Lou L., Liu D., Li X., Proksch P., Yin S., Lin W. (2016). J. Nat. Prod..

[cit64] Li D., Xu Y., Shao C. L., Yang R. Y., Zheng C. J., Chen Y. Y., Fu X. M., Qian P. Y., She Z. G., Voogd N. J., Wang C. Y. (2012). Mar. Drugs.

[cit65] Hu H. Q., Li Y. H., Fan Z. W., Yan W. L., He Z. H., Zhong T. H., Gai Y. B., Yang X. W. (2022). Chem. Biodiversity.

[cit66] Scherlach K., Hertweck C. (2006). Org. Biomol. Chem..

[cit67] Lin Z. J., Zhang G. J., Zhu T. J., Liu R., Wei H. J., Gu Q. Q. (2009). Helv. Chim. Acta.

[cit68] Lv F. Y., Mandi A., Li X. M., Chi L. P., Li X., Wang B. G., Kurtan T., Meng L. H. (2023). Deep Sea Res., Part I.

[cit69] Shigemori H., Wakuri S., Yazawa K., Nakamura T., Sasaki T., Kobayashi J. i. (1991). Tetrahedron.

[cit70] Kito K., Ookura R., Yoshida S., Namikoshi M., Ooi T., Kusumi T. (2008). Org. Lett..

[cit71] Henke M. T. , PhD thesis, Northwestern University, 2016

[cit72] Xu D., Ondeyka J., Harris G. H., Zink D., Kahn J. N., Wang H., Bills G., Platas G., Wang W., Szewczak A. A., Liberator P., Roemer T., Singh S. B. (2011). J. Nat. Prod..

[cit73] Wang W., Zhu T., Tao H., Lu Z., Fang Y., Gu Q., Zhu W. (2007). J. Antibiot..

[cit74] Sumilat D. A., Yamazaki H., Endo K., Rotinsulu H., Wewengkang D. S., Ukai K., Namikoshi M. (2017). J. Nat. Med..

[cit75] Shaaban M., Nasr H., Mohamed T. K., Mahmoud S. F., El-Metwally M. M., Abdelwahab A. B. (2023). Z. Naturforsch., C: J. Biosci..

[cit76] Wu Z. Y., Liu F., Ke S. Y., Zhang Z. G., Hu H. T., Fang W., Xiao S. Y. J., Zhang Y. N., Wang Y. Y., Wang K. M. (2023). Plants.

[cit77] Tibashailwa N., Stephano F., Shadrack D. M., Munissi J. J. E., Nyandoro S. S. (2023). Neurotoxicology.

[cit78] Zhang Y., Jia A., Chen H., Wang M., Ding G., Sun L., Li L., Dai M. (2017). J. Antibiot..

[cit79] Dembitsky V. M., Rezanka T., Shubina E. E. (1993). Phytochemistry.

[cit80] Nagasawa H., Isogai A., Suzuki A., Tamura S. (1979). Agric. Biol. Chem..

[cit81] Li X., Li X. D., Li X. M., Xu G. M., Liu Y., Wang B. G. (2017). RSC Adv..

[cit82] Kralj A., Kehraus S., Krick A., Eguereva E., Kelter G., Maurer M., Wortmann A., Fiebig H. H., Konig G. M. (2006). J. Nat. Prod..

[cit83] Serag A., Salem M. A., Gong S., Wu J. L., Farag M. A. (2023). Metabolites.

[cit84] Salem M. A., Perez de Souza L., Serag A., Fernie A. R., Farag M. A., Ezzat S. M., Alseekh S. (2020). Metabolites.

