# Supplementary material for: Comparative metabolomics study on the secondary metabolites of the red alga, Corallina officinalis and its associated endosymbiotic fungi
Source: RSC Adv. 2024 Jun 20;14(26):18553–66. doi: 10.1039/d4ra01055h (PMC11187739; doi:10.1039/d4ra01055h)
Supplement: RA-014-D4RA01055H-s001 [file RA-014-D4RA01055H-s001.pdf]

Electronic Supplementary Material (ESI) for RSC Advances.
This journal is © The Royal Society of Chemistry 2024

Comparative Metabolomics Study on the Secondary Metabolites of the Red Alga, *Corallina officinalis* and its Associated Endosymbiotic Fungi

Sherif M Shama [a], Ahmed M. Elissawy [b,c,] , Mohamed A. Salem [a], Fadia S. Youssef [b], Mohamed S. Elnaggar [b] , Hesham R. El-Seedi [d,e,f], Shaden A. M. Khalifa[g], Khaled Briki [h], Dalia Ibrahim Hamdan [a] and Abdel Nasser B. Singab[b,c*]

[a] *Department of Pharmacognosy and Natural Products, Faculty of Pharmacy, Menoufia University, Shibin Elkom, 32511, Egypt.*

[b] *Department of Pharmacognosy, Faculty of Pharmacy, Ain-Shams University, Cairo 11566, Egypt.*

[c] *Center of Drug Discovery Research and Development, Ain-Shams University, Cairo 11566, Egypt.*

[d] *International Research Center for Food Nutrition and Safety, Jiangsu University, Zhenjiang 212013, China*

[e] *Pharmacognosy Group, Department of Pharmaceutical Biosciences, Uppsala University, Biomedical Centre, Box 591, SE 751 24 Uppsala, Sweden.*

[f] *International Joint Research Laboratory of Intelligent Agriculture and Agri-Products Processing, Jiangsu Education Department, Jiangsu University, Nanjing 210024, China*

[g] *Psychiatry and Neurology Department, Capio Saint Göran's Hospital, Sankt Göransplan 1, 112 19 Stockholm, Sweden*

[h] *Laboratory of organic chemistry and natural substance, University Ziane Achour, Djelfa, Algeria*

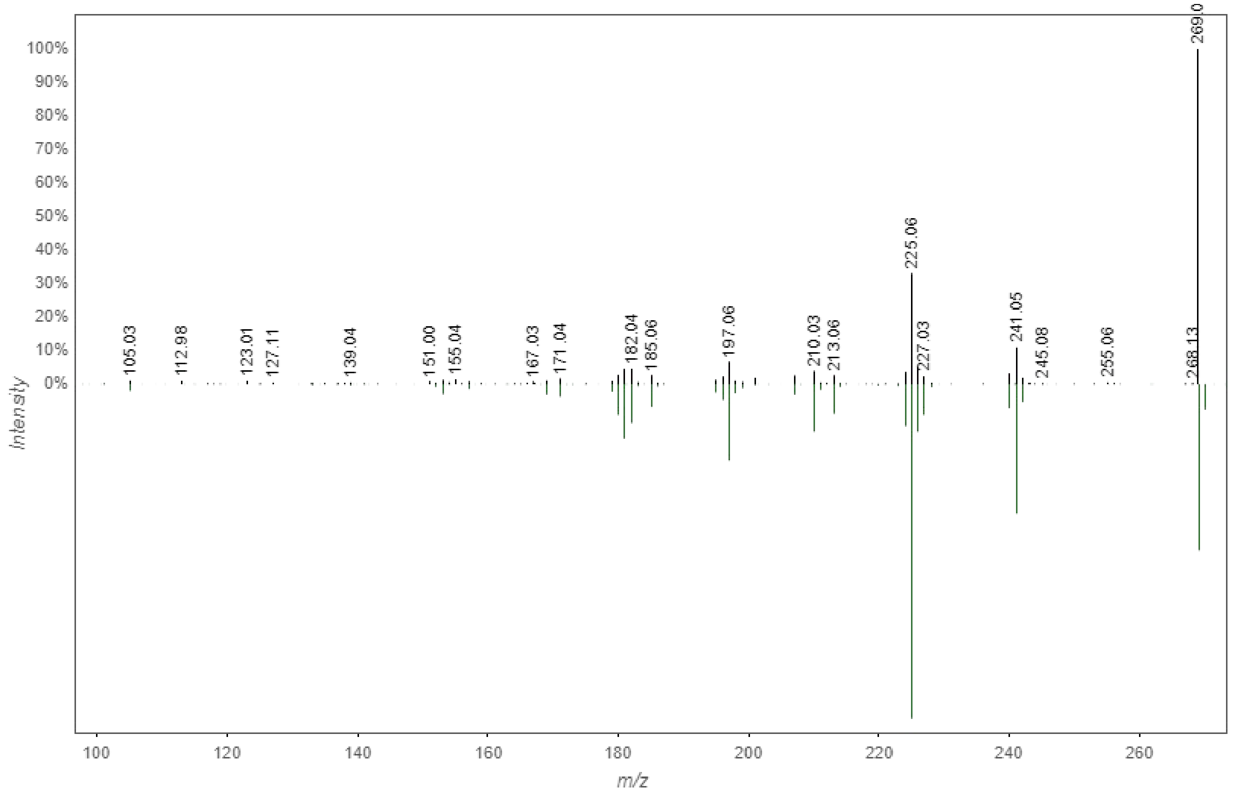

Figure S1. MS/MS spectrum (upper part) mirror matched of with GNPS library (lower part) of Emodin identified from *Aspergillus nidulans* as analyzed by LC/MS in negative ionization mode.

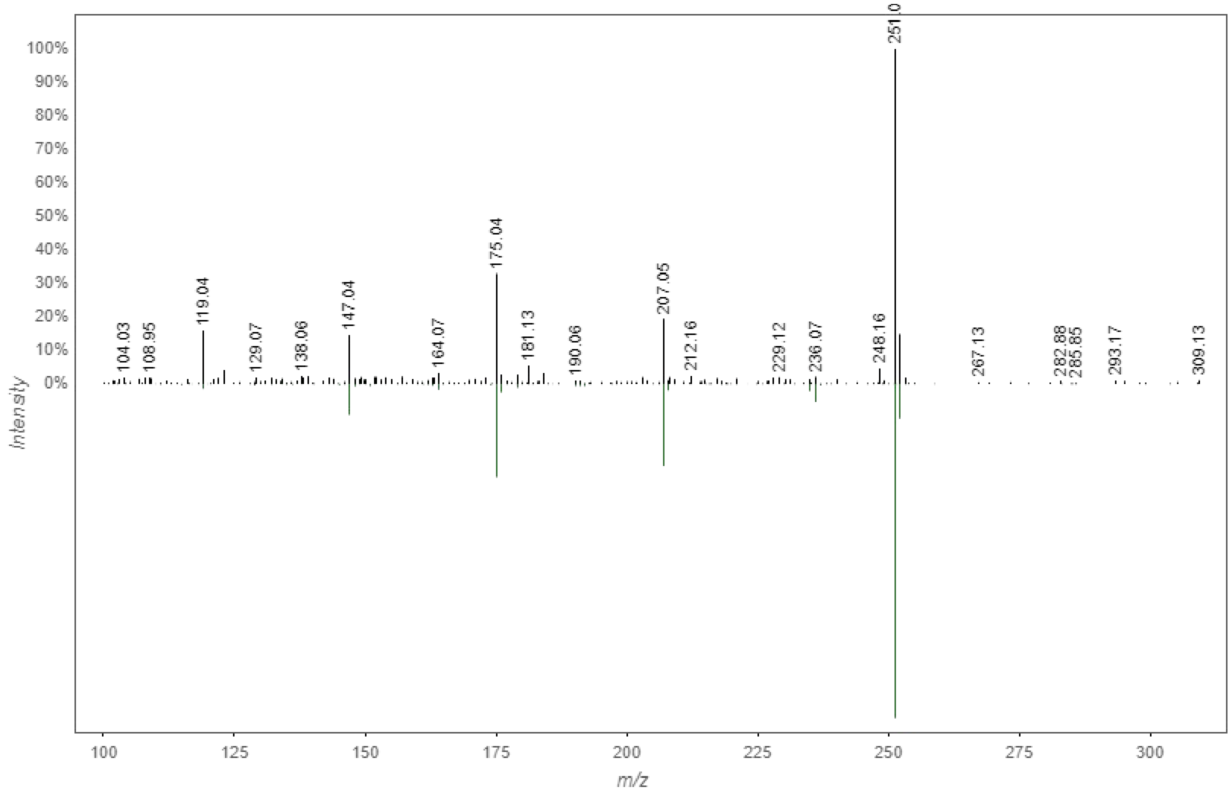

Figure S2. MS/MS spectrum (upper part) mirror matched of with GNPS library (lower part) of Sinapine identified from *Aspergillus flavus* as analyzed by LC/MS in positive ionization mode.

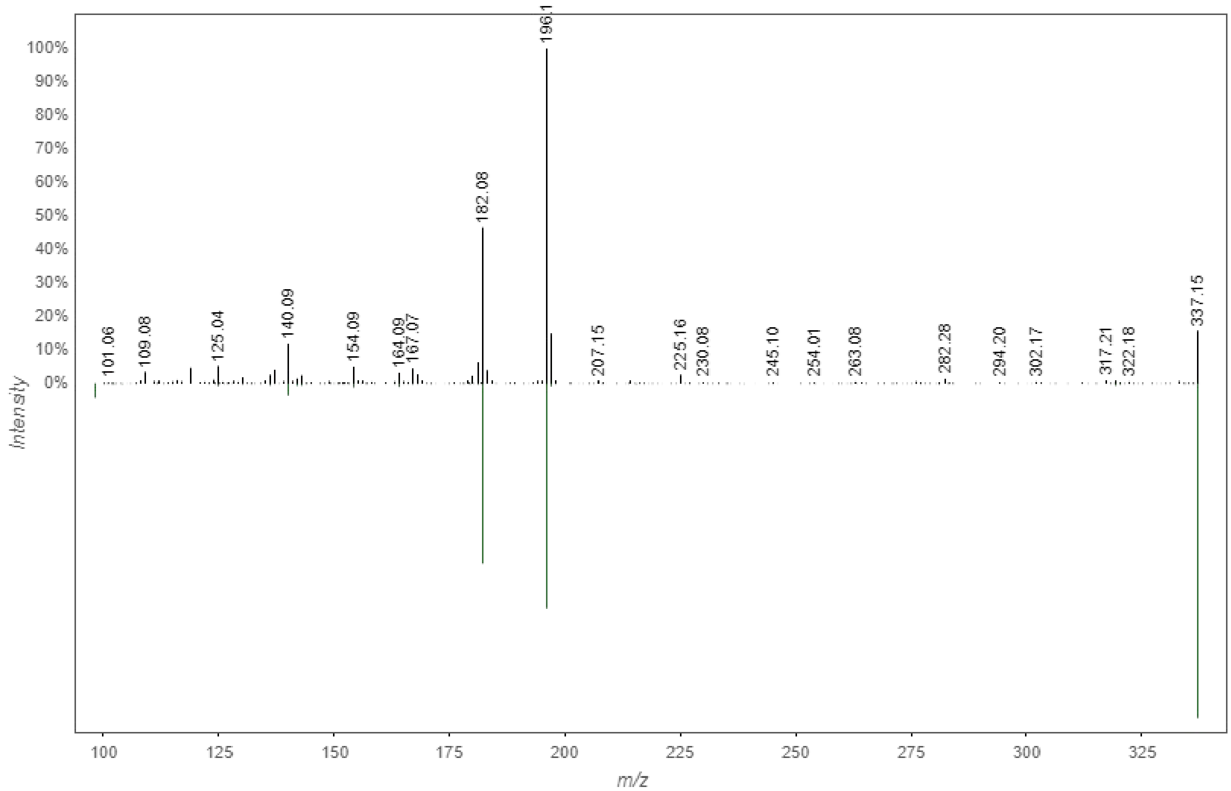

Figure S3. MS/MS spectrum (upper part) mirror matched of with GNPS library (lower part) of Cyclopiazonic acid identified from *Aspergillus flavus* as analyzed by LC/MS in positive ionization mode.

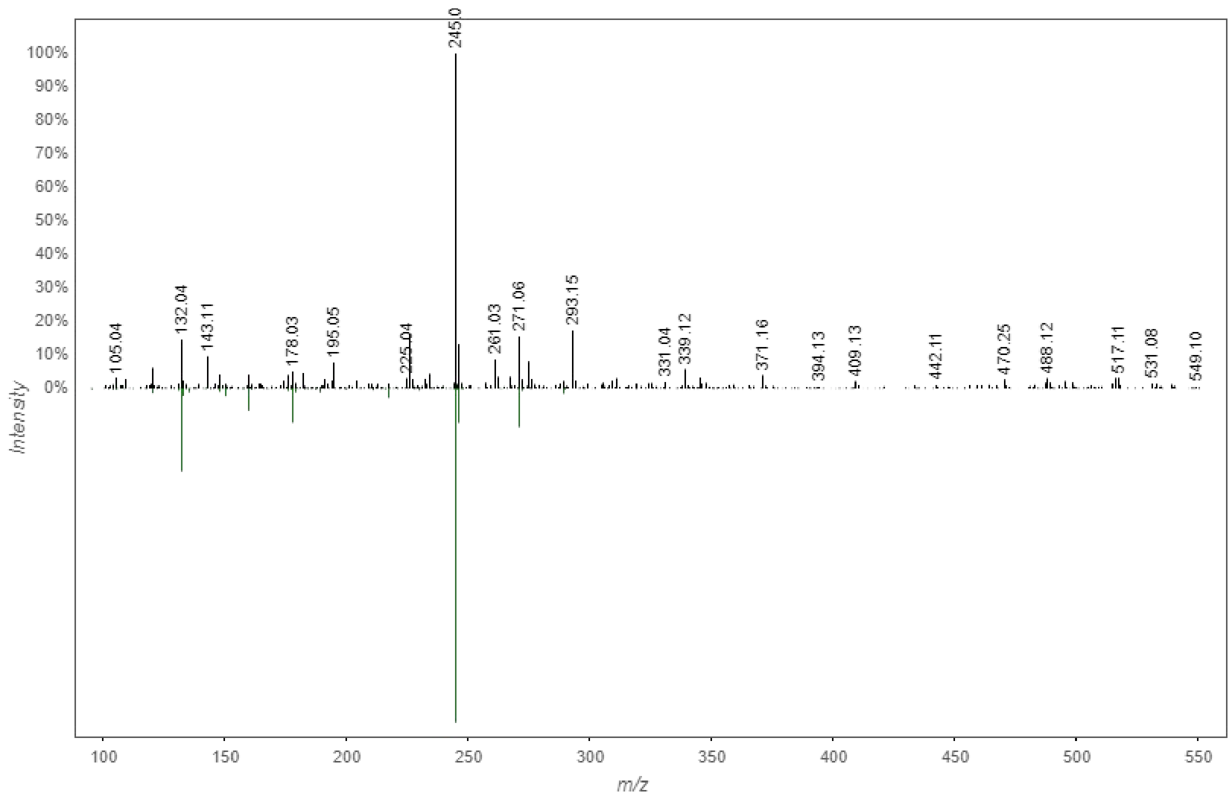

Figure S4. MS/MS spectrum (upper part) mirror matched of with GNPS library (lower part) of Dethiosecoemestrin identified from *Aspergillus nidulans* as analyzed by LC/MS in positive ionization mode.

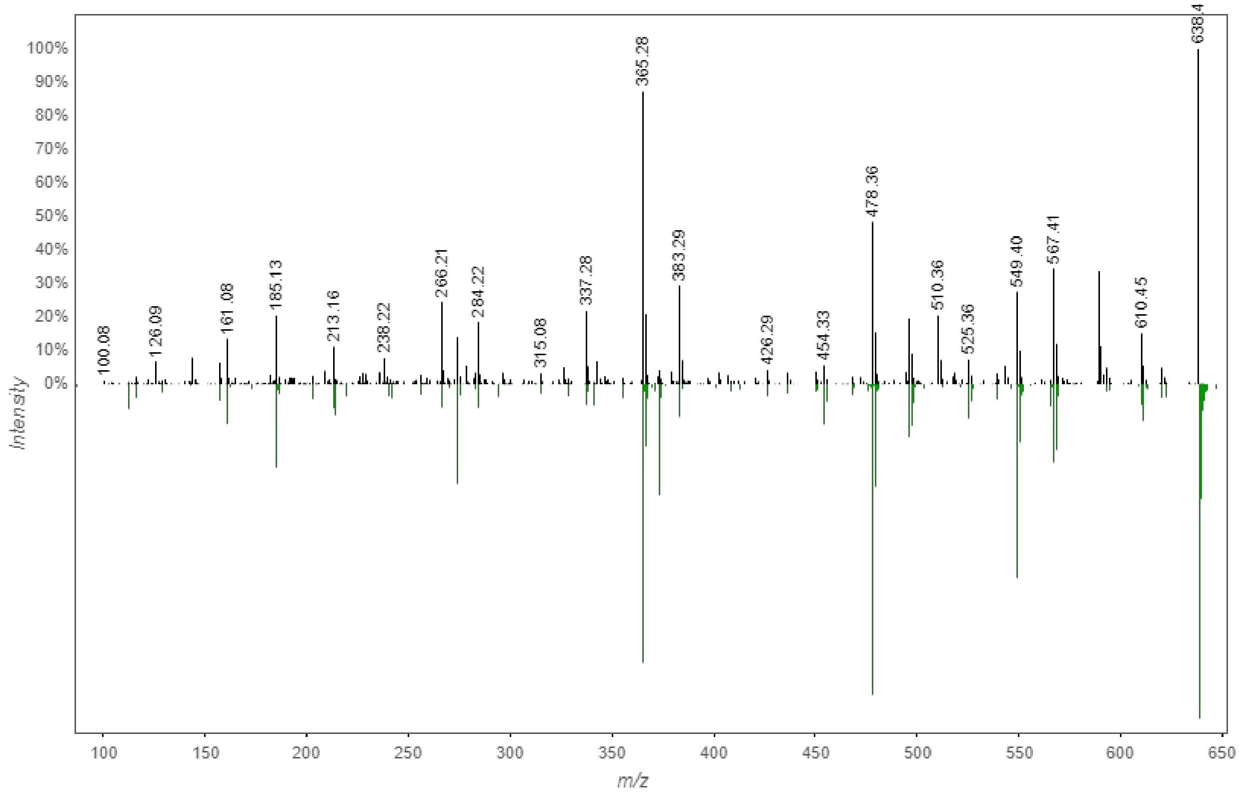

Figure S5. MS/MS spectrum (upper part) mirror matched of with GNPS library (lower part) of Scopularide E identified from *Aspergillus nidulans* as analyzed by LC/MS in positive ionization mode.

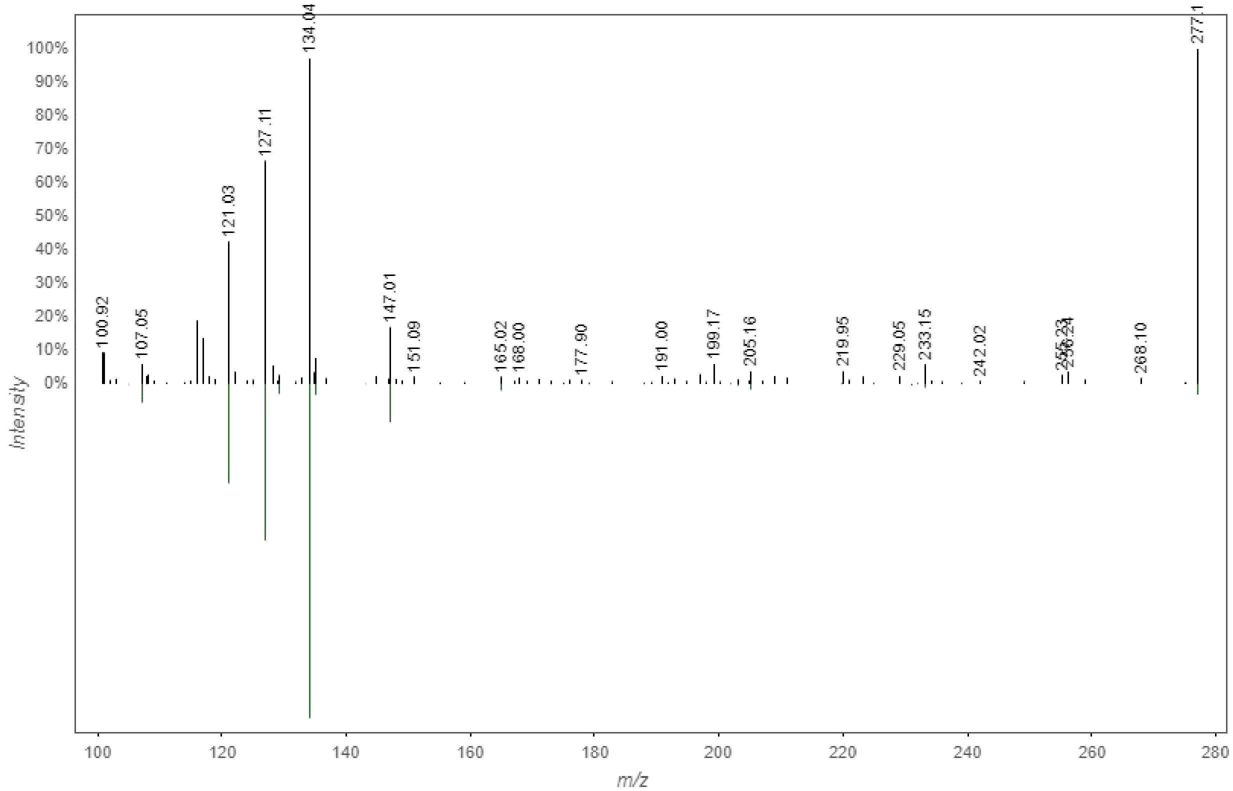

Figure S6. MS/MS spectrum (upper part) mirror matched of with GNPS library (lower part) of 2-(((2-Ethylhexyl) oxy) carbonyl) benzoic acid identified from *Aspergillus flavus* as analyzed by LC/MS in negative ionization mode**.**

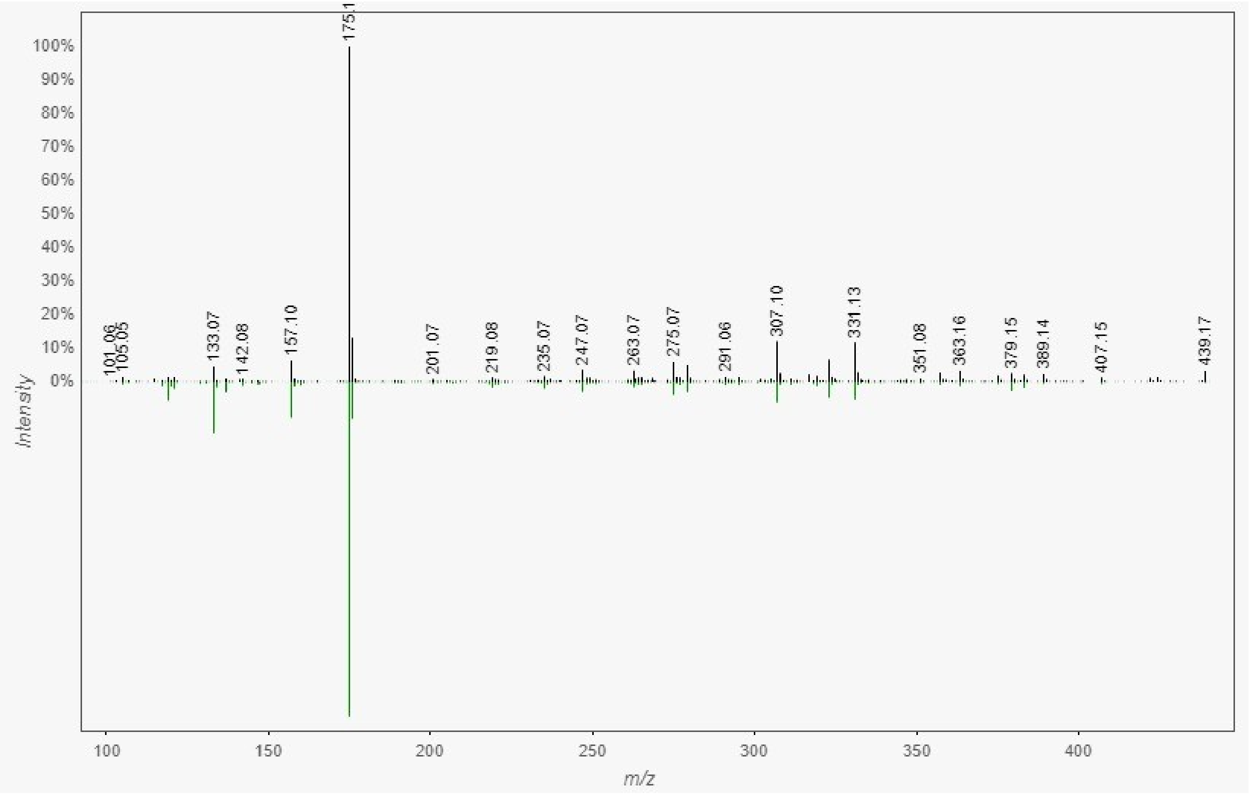

Figure S7. MS/MS spectrum (upper part) mirror matched of with GNPS library (lower part) of Butyrolactone VII identified from *Aspergillus flavipes* as analyzed by LC/MS in positive ionization mode.

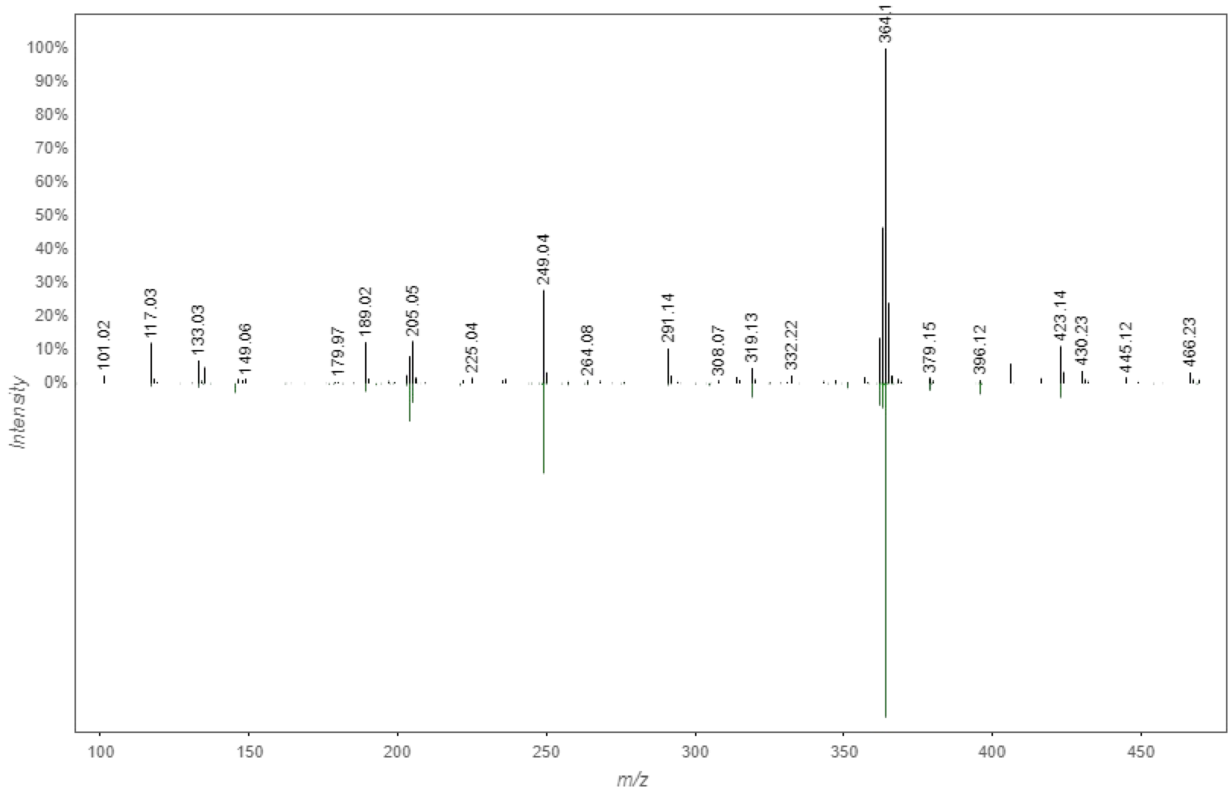

Figure S8. MS/MS spectrum (upper part) mirror matched of with GNPS library (lower part) of Butyrolactone I identified from *Aspergillus flavipes* as analyzed by LC/MS in negative ionization mode.

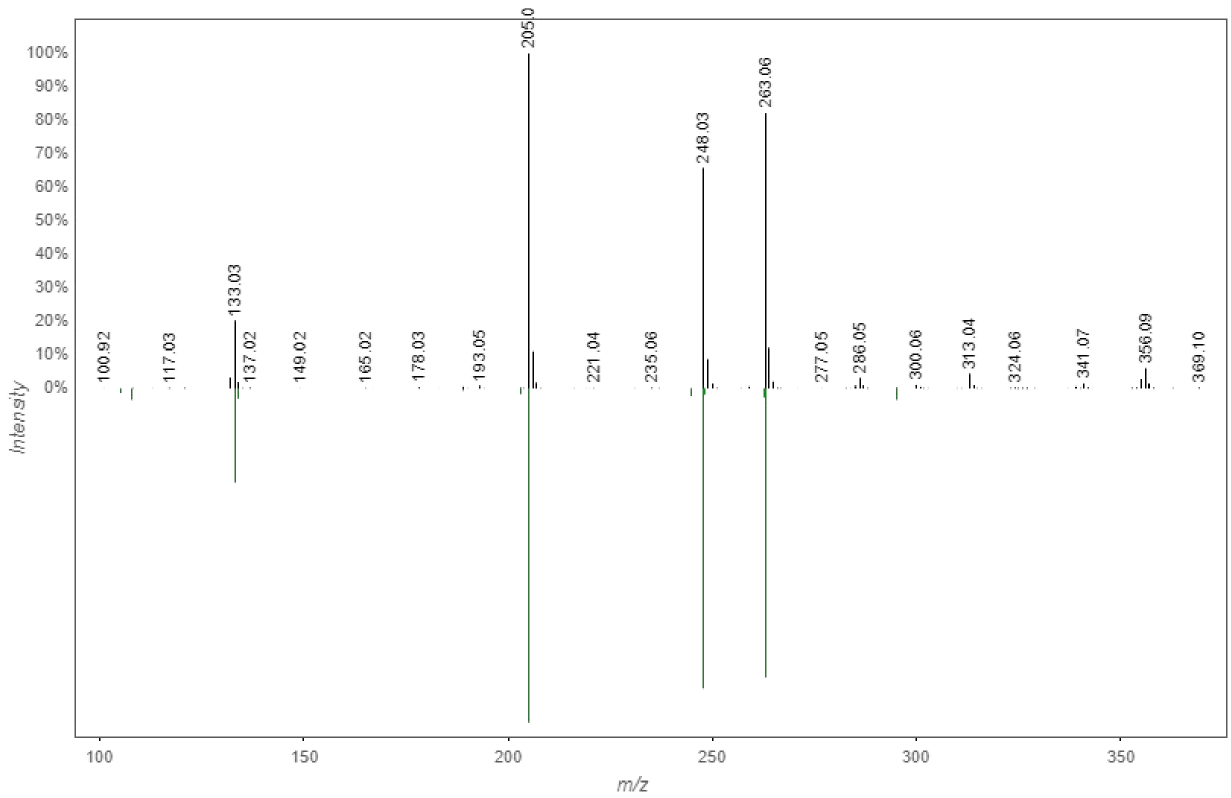

Figure S9. MS/MS spectrum (upper part) mirror matched of with GNPS library (lower part) of 2-O-methylbutyrolactone II identified from *Aspergillus flavipes* as analyzed by LC/MS in negative ionization mode.

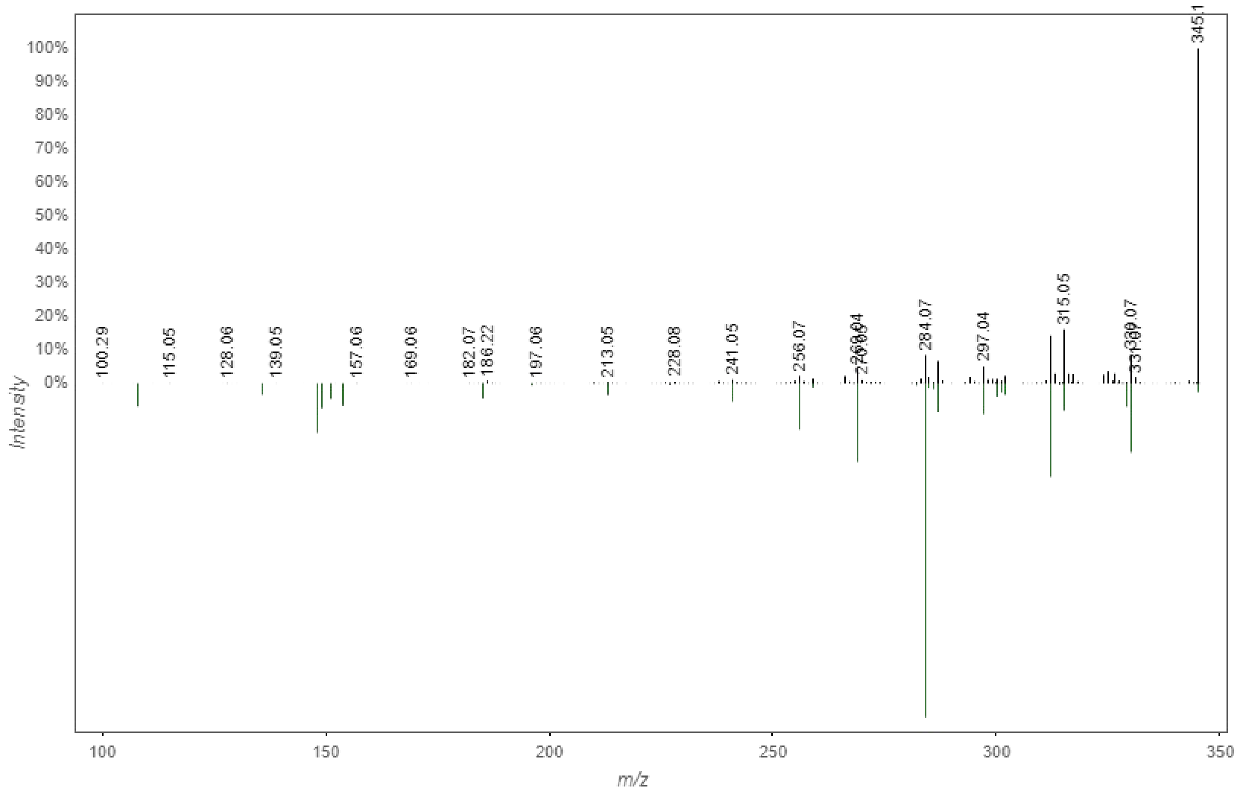

Figure S10. MS/MS spectrum (upper part) mirror matched of with GNPS library (lower part) of 5,2'-Dihydroxy-3,7,8-trimethoxyflavone identified from *Aspergillus flavipes* as analyzed by LC/MS in positive ionization mode.

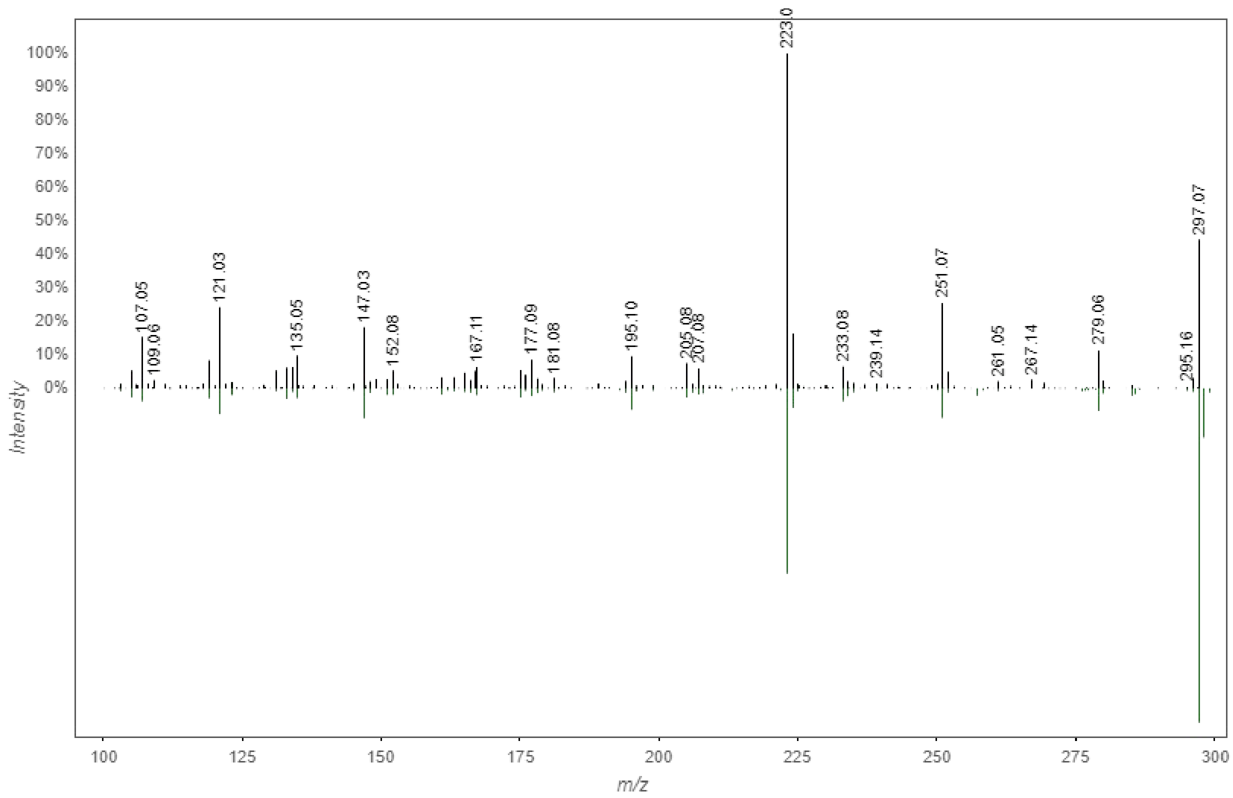

Figure S11. MS/MS spectrum (upper part) mirror matched of with GNPS library (lower part) of Aspulvinone E identified from *Aspergillus flavipes* as analyzed by LC/MS in positive ionization mode.

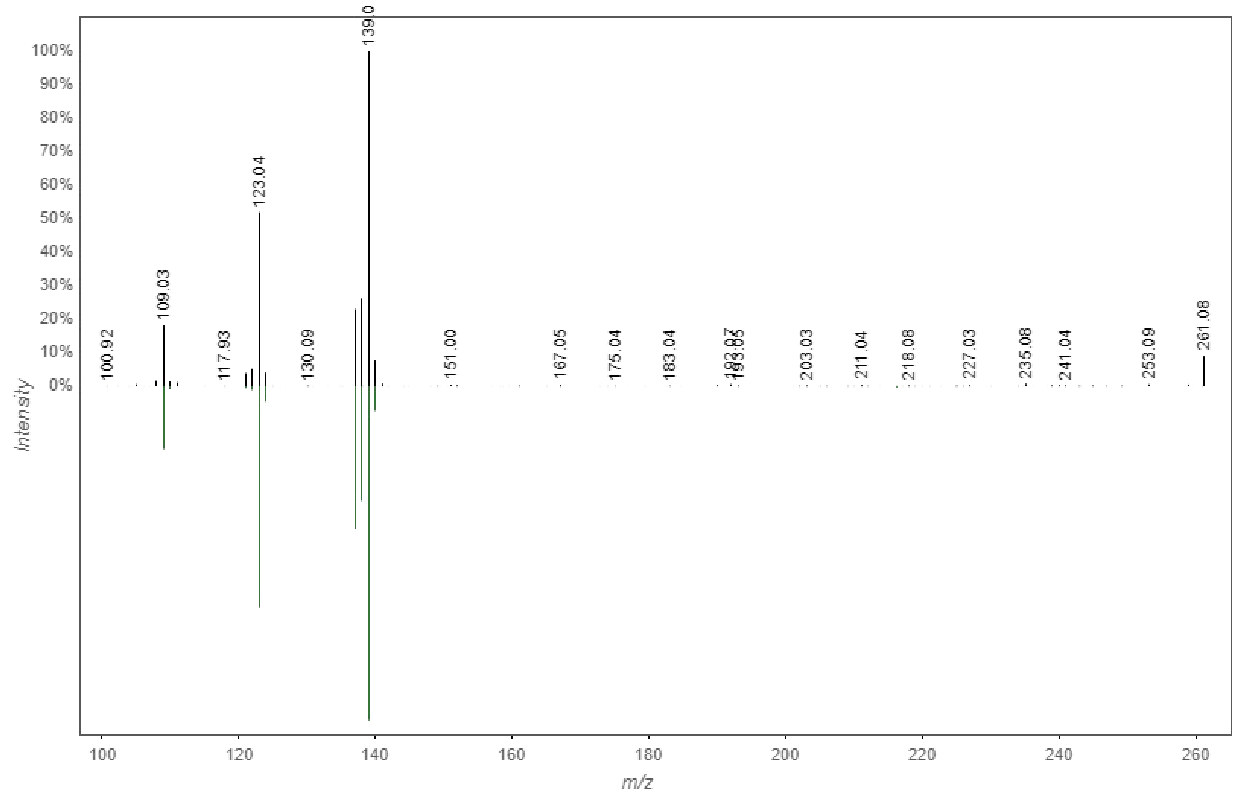

Figure S12. MS/MS spectrum (upper part) mirror matched of with GNPS library (lower part) of violaceol I identified from *Aspergillus nidulans* as analyzed by LC/MS in negative ionization mode.

Table S1. Detailed annotation of metabolites detected in the ethyl acetate extracts of the red alga, *C. officinalis*, and associated Endosymbiotic fungi namely, *A. flavus*, *A. nidulans*, and *A. flavipes* using UPLC-MS/MS Analysis in both positive and negative modes.

Peak no.	tR (min)	Annotated Compound	Adducts	m/z	MS/MS	Elemental Composition	Ref.
1.	4.06	Orsellinic acid	$[M-H]^-$	167.03418	123.0443, 152.0108, 151.0393, 137.0237, 121.0285	$C_8H_8O_4$	[1]
2.	4.14	*p*-hydroxyphenyl acetic acid	$[M+H]^+$	153.0546	131.9639, 113.9639, 105.0702	$C_8H_8O_3$	[2]
3.	4.17	5-hydroxyaverantin	$[M+H]^+$	389.1238	357.0978, 331.1364, 291.1412, 226.8936, 208.8831, 174.9832, 146.9615, 131.9743, 128.9508, 113.9639	$C_{20}H_{20}O_8$	GNPS
4.	5.09	Terrein	$[M-H]^-$	153.05495	151.0392, 123.0442, 135.0442	$C_8H_{10}O_3$	GNPS
5.	5.13	3-methylorsellinicacid	$[M+H]^+$	183.0652	179.0336, 158.0599, 152.0421, 151.0388, 137.0596, 132.0443, 130.0651, 113.9638, 109.0650, 103.0545	$C_9H_{10}O_4$	[3]
6.	5.20	Kojic acid methyl ether	$[M-H]^-$<br>$[M+H]^+$	155.03419<br>157.04964	153.0357, 151.0394, 146.9379, 135.0444, 132.8671, 123.0443	$C_7H_8O_4$	[4]
7.	5.33	Insulicolide A	$[M+H]^+$	432.1655	417.1415, 349.1830, 279.0736, 261.0630, 248.0914, 239.0816, 209.0441, 195.0285, 181.0493, 138.0310, 113.9638	$C_{22}H_{25}NO_8$	[5]

8.	5.45	Orsellinaldehyde	$[M-H]^-$	151.03934	123.0442, 133.0286, 124.0476, 121.0286	$C_8H_8O_3$	[6]
			$[M+H]^+$	153.05469			
9.	5.63	Sinapine	$[M]^+$	310.16489	251.0912, 207.0651, 175.0389, 147.0421, 146.9614, 131.9743, 116.9722, 113.9639	$C_{16}H_{24}NO_5$	GNPS
10.	6.18	N-acetyl-L-leucine	$[M-H]^-$	172.09734	112.9848, 130.0866, 135.0306, 109.0650, 121.0287	$C_8H_{15}NO_3$	GNPS
11.	6.43	Penidiamide	$[M-H]^-$	333.1375	119.0493, 129.0007, 149.0432, 199.0432, 211.1450, 229.0904	$C_{19}H_{18}N_4O_2$	GNPS
12.	6.60	Asperflavin	$[M+H]^+$	289.1069	285.0753, 257.0807, 236.0914, 223.0598, 179.0337, 151.0389, 131.9742, 121.0285, 113.9639, 107.0494	$C_{16}H_{16}O_5$	[7]
13.	6.78	Phenylalanine	$[M-H]^-$	164.0710	147.0444, 134.8646, 132.8671, 116.9276, 112.9847	$C_9H_{11}NO_2$	GNPS
14.	6.92	Speradine D	$[M+H]^+$	387.1551	260.0880, 220.0957, 194.0802, 179.0565, 151.0745, 121.0644	$C_{20}H_{22}N_2O_6$	[8]
15.	6.92	Cichorine	$[M+H]^+$	194.0810	179.0565, 165.0538, 151.0745, 123.0675, 121.0644	$C_{10}H_{11}NO_3$	[9]
16.	7.71	Ferulic acid	$[M-H]^-$	193.0501	178.02520, 176.0450, 149.05910	$C_{10}H_{10}O_4$	[10]
			$[M+H]^+$	195.0651	180.04380, 177.05580, 147.04610		
17.	7.71	Nidulol	$[M+H]^+$	195.0651	189.0541, 180.0412, 165.0542, 162.0308, 151.0386, 147.0437, 137.0593, 119.0491,	$C_{10}H_{10}O_4$	[11]

					109.0649, 107.0493		
18.	7.97	2-O-methyl-9-dehydroxyeurotinone	[M-H]⁻ [M+H]⁺	285.0771 287.0914	135.0444, 147.0444, 214.0873, 179.0345, 119.0493	$C_{16}H_{14}O_5$	[12]
19.	8.36	Asperfuranone	[M+H]⁺	333.1702	241.1539, 206.1408, 191.1174, 169.0968, 152.0941, 137.0707, 110.0476	$C_{19}H_{24}O_5$	[13]
20.	8.40	4-Hydroxyphenylpyruvic acid	[M-H]⁻	179.0341	108.0207, 121.0286, 135.0305, 115.0391	$C_9H_8O_4$	GNPS
21.	8.66	Homogenentisic acid	[M-H]⁻	167.0339	112.9845, 116.9275, 132.8669, 134.8642, 136.8623	$C_8H_8O_4$	GNPS
22.	8.69	Alternariol monomethyl ether	[M-H]⁻	271.0612	269.0699, 228.0429, 207.0298, 176.0349, 148.0398, 137.0237, 122.0366, 107.0368	$C_{15}H_{12}O_5$	[14]
23.	8.96	Daidzein	[M-H]⁻	253.0509	224.0475, 208.0523, 208.0526, 133.0332, 145.0501, 112.9847	$C_{15}H_{10}O_4$	GNPS
24.	9.02	Sterigmatocystin hemiacetal	[M-H]⁻ [[M+H]⁺	341.0673 343.0811	325.0690, 269.0794, 237.0523, 179.0330, 161.0225, 147.0433, 133.0278, 121.0280, 105.0333	$C_{18}H_{14}O_7$	[15]
25.	9.20	Erythroglaucin	[M+Na]⁺	301.0705	295.0965, 287.0540, 257.0801, 231.0642, 194.0807, 179.0698, 165.0544, 151.0385, 123.0439, 109.0284	$C_{16}H_{12}O_6$	[16]
26.	9.22	1-hydroxy-6-methyl-8-hydroxymethylxanthone	[M+H]⁺	257.0807	239.0695, 229.0853, 211.0753, 206.0806, 201.0904, 193.0491, 169.0643, 165.0543,	$C_{15}H_{12}O_4$	GNPS

					152.0616, 141.0695, 121.0283, 115.0542, 107.0492		
27.	9.32	2,3,6,8,9-pentahydroxy-1-oxo-3-(2-oxopropyl)-1,2,3,4-tetra-hydroanthracene-2-carboxylic acid	$[M-H]^-$ $[M+H]^+$	375.0727 377.0865	376.1639, 364.2257, 354.2414, 344.2206, 340.2262, 285.0746, 269.1525, 257.0797, 220.1686, 138.0908, 133.0853, 119.0853, 113.9635	$C_{18}H_{16}O_9$	GNPS
28.	9.39	Sterigmatocystin	$[M-H]^-$ $[M+H]^+$	323.0564 325.0707	307.0585, 279.0637, 251.0690, 223.0589, 161.0225, 133.0278, 123.0800, 107.0490	$C_{18}H_{12}O_6$	[15, 17, 18]
29.	9.45	Atrochryson carboxylic acid	$[M-H]^-$ $[M+H]^+$	317.0665 319.0813	286.0486, 300.0640, 379.1588, 116.927, 253.2539, 183.0116	$C_{16}H_{14}O_7$	GNPS
30.	9.70	Aspergilloid C	$[M-H]^-$	279.1242	217.1234, 177.0916, 147.0444, 112.9847	$C_{15}H_{20}O_5$	[19]
31.	9.77	Violaceol i	$[M-H]^-$	261.0773	139.0393, 261.0772, 207.0298, 123.0443, 109.0343	$C_{14}H_{14}O_5$	GNPS
32.	9.80	Versiquinazoline E	[M+H]+	444.1651	401.1199, 376.1382, 354.1332, 338.1007, 313.1422, 271.0598, 264.1225, 206.0806, 193.0492, 179.0544, 165.0544, 151.0749, 113.9637	$C_{24}H_{21}N_5O_4$	[20]
33.	9.93	Aspulvinone E	$[M+H]^+$	297.0759	279.0640, 251.0693, 233.0845, 223.0745, 205.0821, 195.1042, 177.0935, 147.0433, 121.0743, 107.0491	$C_{17}H_{12}O_5$	GNPS

34.	10.02	Aspergiterpenoid A	$[M+H]^+$	237.1848	207.1488, 191.1176, 177.1018, 165.1020, 163.0863, 137.0708, 120.0808, 113.9638	$C_{15}H_{24}O_2$	[21]
35.	10.08	Epicoccolide B	$[M+H]^+$	359.0761	341.0686, 313.0690, 297.0743, 285.0742, 251.0690, 223.0743, 147.0433, 121.0280	$C_{18}H_{14}O_8$	[22]
36.	10.20	5-methoxydihydrosterigmatocystin	$[M-H]^-$ $[M+H]^+$	355.0822 357.0966	339.0843, 325.0682, 269.0791, 223.0744, 181.0487, 147.0432, 131.0277, 121.0279	$C_{19}H_{16}O_7$	[15]
37.	10.87	Alternariol	$[M-H]^-$	257.0455	245.0822, 237.0924, 213.0556, 207.0298, 143.0495, 139.0394, 137.0236, 122.0365	$C_{14}H_{10}O_5$	[14]
38.	11.33	Curvularin	$[M-H]^-$	291.1236	115.0755, 179.0344, 270.1350, 123.0442	$C_{16}H_{20}O_5$	GNPS
39.	11.96	Aspoquinoline C	$[M-H]^-$	482.2176	468.2398, 426.2294, 418.2608, 300.0643, 263.0564, 208.0977, 179.0344, 142.0344	$C_{27}H_{33}NO_7$	[23]
40.	12.12	Cytochalasin Z17	$[M+H]^+$	464.2438	456.2343, 450.2233, 402.2633, 384.2528, 364.1887, 303.0851, 206.1170, 166.0857, 135.0800, 107.0491	$C_{28}H_{33}NO_5$	[24]
41.	12.35	Dethiosecoemestrin	$[M+NH_4]^+$	550.1457	488.1190, 319.0624, 293.1484, 271.0591, 245.0549, 226.2156, 179.0695, 178.0321, 132.0440	$C_{27}H_{20}N_2O_{10}$	GNPS
42.	12.58	Emestrin	$[M+H]^+$	599.0783	594.3244, 580.3612, 579.3527, 578.3495, 517.1217, 499.1112, 489.1264, 471.1168,	$C_{27}H_{22}N_2O_{10}S_2$	[25]

					443.1211, 415.1260, 386.1022, 285.1106, 257.0542, 229.0596, 201.0647, 132.0437, 115.0494		
43.	12.67	Cytochalasin z8	$[M+H]^+$	466.2593	448.2463, 430.2358, 366.2043, 356.1475, 303.0846, 217.0484, 166.0854, 120.0804	$C_{28}H_{35}NO_5$	[24]
44.	12.70	Fellutamide A	$[M-H]^-$ $[M+H]^+$	570.3521 572.3651	534.1860, 518.2125, 474.2226, 416.2194, 356.1471, 290.1520, 240.1367, 217.0481, 120.0802	$C_{27}H_{49}N_5O_8$	[26]
45.	12.74	Acyl-hemiacetal sterigmatocystin	$[M+H]^+$	385.0918	343.0800, 325.0694, 310.0459, 297.0748, 248.1270, 167.0332, 113.9635	$C_{20}H_{16}O_8$	[15]
46.	12.80	Butyrolactone I	$[M-H]^-$	423.1449	364.1318, 319.1334, 249.0442, 205.0523,179.0344, 133.0313	$C_{24}H_{24}O_7$	GNPS
47.	13.27	Butyrolactone VII	$[M+H]^+$	439.1755	422.2278, 331.1309, 307.0945, 275.0697, 217.0482, 175.1107, 166.0853, 157.1023, 133.0713	$C_{25}H_{26}O_7$	GNPS
48.	13.27	2-O-methylbutyrolactone II	$[M-H]^-$	369.0977	341.0669, 311.1691, 291.2005, 263.0624, 255.2333, 248.0335, 205.0122, 183.0116, 133.0326	$C_{20}H_{18}O_7$	GNPS
49.	13.32	Aspergillide E	$[M+H]^+$	466.22	347.0514, 325.0695, 310.0460, 247.1434, 281.0435, 282.0490, 182.0441, 195.0761, 219.1485	$C_{25}H_{29}N_4O_5$	[27]

50.	13.40	Emericellamide C	$[M+H]^+$	596.4021	568.4059, 554.2728, 547.3453, 525.3638, 454.3265, 341.2424, 325.0686, 291.1694, 266.0648, 244.1647, 226.1541, 179.0697, 159.0912, 130.0648, 123.1166	$C_{30}H_{53}N_5O_7$	[28]
51.	13.45	Fellutamide D	$[M-H]^-$<br>$[M+H]^+$	598.38153<br>600.39789	582.3835, 532.2279, 456.2161, 357.1767, 325.0664, 303.0862, 289.0688, 198.2206,166.0855	$C_{29}H_{53}N_5O_8$	[29]
52.	13.49	Emodin	$[M-H]^-$	269.04626	241.0509, 227.0331, 225.0558, 213.0642, 210.0304, 203.0712, 197.0621, 185.0641, 182.0441, 177.0190, 171.0423, 155.0455, 116.9276, 105.0334	$C_{15}H_{10}O_5$	GNPS
53.	13.49	Eurotinone	$[M+H]^+$	289.07068	288.0609, 243.0635, 201.1624, 166.0851	$C_{15}H_{12}O_6$	[30]
54.	13.49	Monodictyphenone	$[M+H]^+$	289.07068	201.1636, 169.9771, 154.9900, 146.9801, 144.9820, 139.9877, 110.0202, 113.9638	$C_{15}H_{12}O_6$	[31]
55.	13.72	Cyclopiazonic acid	$[M+H]^+$	337.15463	337.1502, 216.0650, 196.1117, 182.0807, 140.0703	$C_{20}H_{20}N_2O_3$	[32]
56.	13.76	Sydonic acid	$[M-H]^-$	265.14838	263.0565, 248.0328, 205.0139, 179.0344	$C_{15}H_{22}O_4$	[33]
57.	13.92	Emericellamide A	$[M+H]^+$	610.41785	582.4222, 561.3615, 539.3798, 468.3429, 441.2694, 355.2584, 330.0964, 325.0697, 301.0336, 250.0703, 216.0650, 184.0729,	$C_{31}H_{55}N_5O_7$	[28]

					179.0729, 130.0649, 123.1167		
58.	13.99	Asperphenamate	[M+H]+	507.22897	456.2124, 303.0862, 289.0686, 256.1317, 238.1213, 117.0695, 105.0333	$C_{32}H_{30}N_2O_4$	[34]
59.	13.99	2-(((2-Ethylhexyl)oxy)carbonyl)benzoic acid	$[M-H]^-$	277.14456	255.2333, 183.0117, 265.1483, 152.9951, 147.0135, 134.0365, 127.1119, 116.9276, 107.0542	$C_{16}H_{22}O_4$	GNPS
60.	14.08	2-methyleurotinone	$[M-H]^-$<br>$[M+H]^+$	301.0718<br>303.0862	286.0485, 271.0252, 243.0300	$C_{16}H_{14}O_6$	[35]
61.	14.11	Emericellamide E	$[M+H]^+$	624.4332	502.3316, 437.1952, 404.3529, 284.3313, 184.0734, 184.0734, 143.1180, 105.0703	$C_{32}H_{57}N_5O_7$	[28]
62.	14.15	1-hexadecanoyl glycerophosphocholine	$[M+FA-H]^-$	540.3314	480.3103, 311.1691, 291.2005, 255.2333, 183.0116	$C_{24}H_{50}NO_7P$	GNPS
63.	14.22	8-Hydroxy-9,12-octadecadienoic acid	$[M-H]^-$	295.2285	262.1454, 183.0117, 116.9276, 195.1387, 277.2177, 281.2491, 279.2334	$C_{18}H_{32}O_3$	GNPS
64.	14.25	6,8-O-dimethylaverantin	$[M-H]^-$	399.1458	389.0984, 354.0748, 326.0796, 370.1015, 384.1218	$C_{22}H_{24}O_7$	GNPS
65.	14.25	5,2'-Dihydroxy-3,7,8-trimethoxyflavone	$[M+H]^+$	345.0966	345.1021, 331.0744, 330.0708, 325.0655, 315.0655, 315.0475, 312.0605, 297.0437, 287.0527, 284.0657, 270.0424, 256.0721	$C_{18}H_{16}O_7$	GNPS
66.	14.26	Hormonemate F	$[M-H]^-$	607.3275	473.2845, 334.1821, 311.1695, 281.2492,	$C_{29}H_{52}O_{13}$	GNPS

					262.1456, 255.2334, 221.1547, 183.0118, 152.9952, 116.9276		
67.	14.35	Flavoglaucin	$[M+H]^+$	305.2105	313.2734, 277.1797, 184.0732, 137.0596, 122.0965, 111.0443, 104.1073	$C_{19}H_{28}O_3$	[16]
68.	14.37	Scopularide E	$[M+H]^+$	638.4491	610.4521, 589.3916, 567.4099, 549.3994, 525.3612, 478.3625, 454.3312, 383.2890, 365.2784, 352.1892, 337.2801, 266.2103, 216.0646, 213.1602, 185.1334, 161.0821	$C_{33}H_{59}N_5O_7$	GNPS
69.	14.56	Aversin	$[M-H]^-$<br>$[M+H]^+$	367.0818<br>369.0964	345.0954, 303.0850, 289.0687, 184.0725	$C_{20}H_{16}O_7$	[18]
70.	14.5	7-hydroxy-8,14-dimethyl-9-hexadecanoic acid	$[M-H]^-$	297.2439	281.2491, 255.2334, 183.0117, 152.9952, 116.9276	$C_{18}H_{34}O_3$	[36]
71.	14.69	Isoechinulins C	$[M+H]^+$	406.2121	383.2023, 352.1899, 324.3251, 284.3303, 236.1485, 216.0650, 199.0622, 184.0728, 133.0856	$C_{24}H_{27}N_3O_3$	[37]
72.	14.91	Wentinoid A	$[M+H]^+$	349.2370	340.3927, 291.2309, 247.2048, 216.0649, 199.0621, 184.0728, 177.1117, 165.0903, 133.0856, 121.0646, 109.1012	$C_{21}H_{32}O_4$	[38]

73.	15.62	Glycerol linoleate	$[M+H]^+$	355.2815	277.1795, 239.1481, 184.0730, 177.1120, 133.0857, 121.9661, 105.0700	$C_{21}H_{38}O_4$	[4]
74.	15.65	Arugosin G	$[M-H]^-$	491.2434	286.0486, 300.0640, 379.1588, 116.927, 253.2539, 183.0116	$C_{30}H_{36}O_6$	[39]
75.	15.6	Linoleic acid	$[M+H]^+$	281.2474	280.2625, 276.2678, 231.1376, 184.0729, 171.1374, 144.0804, 133.0856, 123.9642, 121.9660, 113.9636, 109.1012	$C_{18}H_{32}O_2$	[4]
76.	16.22	Versiquinazoline (J)	$[M-H]^-$	382.1772	381.1746, 225.2334, 134.8938, 116.9275, 115.9198, 100.9249	$C_{21}H_{25}N_3O_4$	[20]

References:

1. M. V. Nguyen, J. W. Han, H. Kim and G. J. Choi, *Acs Omega*, 2022, **7**, 33273-33279.
2. W. J. Zuo, P. F. Jin, W. H. Dong, H. F. Dai and W. L. Mei, *Chin J Nat Med*, 2014, **12**, 151-153.
3. G. D. Chen, D. Hu, M. J. Huang, J. Tang, X. X. Wang, J. Zou, J. Xie, W. G. Zhang, L. D. Guo, X. S. Yao, I. Abe and H. Gao, *Chemical Communications*, 2020, **56**, 4607-4610.
4. E. M. Eliwa, M. M. El-Metwally, A. H. Halawa, A. M. El-Agrody, A. H. Bedair and M. Shaaban, *Journal of Atoms and Molecules*, 2017, **7**, 1045.
5. L. Rahbaek, C. Christophersen, J. Frisvad, H. S. Bengaard, S. Larsen and B. R. Rassing, *Journal of Natural Products*, 1997, **60**, 811-813.
6. H. Han, C. Yu, J. Qi, P. Wang, P. Zhao, W. Gong, C. Xie, X. Xia and C. Liu, *Microb Cell Fact*, 2023, **22**, 60.
7. X. Yang, M. C. Kang, Y. Li, E. A. Kim, S. M. Kang and Y. J. Jeon, *Molecules*, 2017, **22**, 1823.
8. V. Uka, G. G. Moore, N. Arroyo-Manzanares, D. Nebija, S. De Saeger and J. Diana Di Mavungu, *Toxins (Basel)*, 2017, **9**, 35.
9. J. F. Sanchez, R. Entwistle, D. Corcoran, B. R. Oakley and C. C. Wang, *Medchemcomm*, 2012, **3**, 997-1002.
10. D. O. Abonyi, P. M. Eze, C. C. Abba, N. T. Ujam, P. Proksch, F. B. Okoye and C. O. Esimone, 2018, **8**, 157-167.
11. P. Aukamp and C. Holzapfel, *South African Journal of Chemistry*, 1968, **21**, 26-32.
12. H. J. Yan, X. M. Li, C. S. Li and B. G. Wang, *Helvetica Chimica Acta*, 2012, **95**, 163-168.
13. C. C. Wang, Y. M. Chiang, M. B. Praseuth, P. L. Kuo, H. L. Liang and Y. L. Hsu, *Basic Clin Pharmacol Toxicol*, 2010, **107**, 583-589.
14. K. Kong, Z. Huang, S. Shi, W. Pan and Y. Zhang, *BMC Microbiol*, 2023, **23**, 30.
15. F. Song, B. Ren, C. Chen, K. Yu, X. Liu, Y. Zhang, N. Yang, H. He, X. Liu, H. Dai and L. Zhang, *Appl Microbiol Biotechnol*, 2014, **98**, 3753-3758.
16. V. V. Volkov and C. C. Perry, *Dyes and Pigments*, 2021, **195**, 109719.
17. S. S. Zhang, A. O. Zhu, X. Bai, H. J. Zhu and F. Cao, *Chemistry of Natural Compounds*, 2020, **56**, 964-967.
18. T. Q. Cao, Z. Liu, L. Dong, H. Lee, W. Ko, L. B. Vinh, N. Q. Tuan, Y. C. Kim, J. H. Sohn, J. H. Yim, D. S. Lee and H. Oh, *Molecules*, 2022, **27**, 2851.
19. Z. Liu, J. Y. Zhao, S. F. Sun, Y. Li, J. Qu, H. T. Liu and Y. B. Liu, *J Nat Prod*, 2019, **82**, 1063-1071.
20. Z. Cheng, L. Lou, D. Liu, X. Li, P. Proksch, S. Yin and W. Lin, *J Nat Prod*, 2016, **79**, 2941-2952.
21. D. Li, Y. Xu, C. L. Shao, R. Y. Yang, C. J. Zheng, Y. Y. Chen, X. M. Fu, P. Y. Qian, Z. G. She, N. J. Voogd and C. Y. Wang, *Mar Drugs*, 2012, **10**, 234-241.
22. H. Q. Hu, Y. H. Li, Z. W. Fan, W. L. Yan, Z. H. He, T. H. Zhong, Y. B. Gai and X. W. Yang, *Chem Biodivers*, 2022, **19**, e202100804.
23. K. Scherlach and C. Hertweck, *Org Biomol Chem*, 2006, **4**, 3517-3520.
24. Z. J. Lin, G. J. Zhang, T. J. Zhu, R. Liu, H. J. Wei and Q. Q. Gu, *Helvetica Chimica Acta*, 2009, **92**, 1538-1544.
25. F. Y. Lv, A. Mandi, X. M. Li, L. P. Chi, X. Li, B. G. Wang, T. Kurtan and L. H. Meng, *Deep-Sea Res Pt I*, 2023, **195**, 104004.
26. H. Shigemori, S. Wakuri, K. Yazawa, T. Nakamura, T. Sasaki and J. i. Kobayashi, *Tetrahedron*, 1991, **47**, 8529-8534.
27. K. Kito, R. Ookura, S. Yoshida, M. Namikoshi, T. Ooi and T. Kusumi, *Org Lett*, 2008, **10**, 225-228.
28. M. T. Henke, Northwestern University, 2016.
29. D. Xu, J. Ondeyka, G. H. Harris, D. Zink, J. N. Kahn, H. Wang, G. Bills, G. Platas, W. Wang, A. A. Szewczak, P. Liberator, T. Roemer and S. B. Singh, *J Nat Prod*, 2011, **74**, 1721-1730.

30. W. Wang, T. Zhu, H. Tao, Z. Lu, Y. Fang, Q. Gu and W. Zhu, *J Antibiot (Tokyo)*, 2007, **60**, 603-607.
31. D. A. Sumilat, H. Yamazaki, K. Endo, H. Rotinsulu, D. S. Wewengkang, K. Ukai and M. Namikoshi, *J Nat Med*, 2017, **71**, 776-779.
32. M. Shaaban, H. Nasr, T. K. Mohamed, S. F. Mahmoud, M. M. El-Metwally and A. B. Abdelwahab, *Z Naturforsch C J Biosci*, 2023, **78**, 149-156.
33. Z. Y. Wu, F. Liu, S. Y. Ke, Z. G. Zhang, H. T. Hu, W. Fang, S. Y. J. Xiao, Y. N. Zhang, Y. Y. Wang and K. M. Wang, *Plants-Basel*, 2023, **12**, 203.
34. N. Tibashailwa, F. Stephano, D. M. Shadrack, J. J. E. Munissi and S. S. Nyandoro, *Neurotoxicology*, 2023, **94**, 147-157.
35. Y. Zhang, A. Jia, H. Chen, M. Wang, G. Ding, L. Sun, L. Li and M. Dai, *J Antibiot (Tokyo)*, 2017, **70**, 1138-1141.
36. V. M. Dembitsky, T. Rezanka and E. E. Shubina, *Phytochemistry*, 1993, **34**, 1057-1059.
37. H. Nagasawa, A. Isogai, A. Suzuki and S. Tamura, *Agricultural and Biological Chemistry*, 1979, **43**, 1759-1763.
38. X. Li, X. D. Li, X. M. Li, G. M. Xu, Y. Liu and B. G. Wang, *Rsc Advances*, 2017, **7**, 4387-4394.
39. A. Kralj, S. Kehraus, A. Krick, E. Eguereva, G. Kelter, M. Maurer, A. Wortmann, H. H. Fiebig and G. M. Konig, *J Nat Prod*, 2006, **69**, 995-1000.
